# Prevalence, Specificity and Determinants of Lipid-Interacting PDZ Domains from an In-Cell Screen and *In Vitro* Binding Experiments

**DOI:** 10.1371/journal.pone.0054581

**Published:** 2013-02-04

**Authors:** Ylva Ivarsson, Anna Maria Wawrzyniak, Rudra Kashyap, Jolanta Polanowska, Stéphane Betzi, Frédérique Lembo, Elke Vermeiren, Driss Chiheb, Nicolas Lenfant, Xavier Morelli, Jean-Paul Borg, Jérôme Reboul, Pascale Zimmermann

**Affiliations:** 1 Department of Human Genetics, K. U. Leuven, Leuven, Belgium; 2 Inserm, U1068, CRCM, Marseille, France; 3 Institut Paoli-Calmettes, Marseille, France; 4 Université Aix-Marseille, Marseille, France; 5 CNRS, UMR7258, CRCM, Marseille, France; Institute of Biology Valrose, France

## Abstract

**Background:**

PDZ domains are highly abundant protein-protein interaction modules involved in the wiring of protein networks. Emerging evidence indicates that some PDZ domains also interact with phosphoinositides (PtdInsPs), important regulators of cell polarization and signaling. Yet our knowledge on the prevalence, specificity, affinity, and molecular determinants of PDZ-PtdInsPs interactions and on their impact on PDZ-protein interactions is very limited.

**Methodology/Principal Findings:**

We screened the human proteome for PtdInsPs interacting PDZ domains by a combination of *in vivo* cell-localization studies and *in vitro* dot blot and Surface Plasmon Resonance (SPR) experiments using synthetic lipids and recombinant proteins. We found that PtdInsPs interactions contribute to the cellular distribution of some PDZ domains, intriguingly also in nuclear organelles, and that a significant subgroup of PDZ domains interacts with PtdInsPs with affinities in the low-to-mid micromolar range. *In vitro* specificity for the head group is low, but with a trend of higher affinities for more phosphorylated PtdInsPs species. Other membrane lipids can assist PtdInsPs-interactions. PtdInsPs-interacting PDZ domains have generally high pI values and contain characteristic clusters of basic residues, hallmarks that may be used to predict additional PtdInsPs interacting PDZ domains. In tripartite binding experiments we established that peptide binding can either compete or cooperate with PtdInsPs binding depending on the combination of ligands.

**Conclusions/Significance:**

Our screen substantially expands the set of PtdInsPs interacting PDZ domains, and shows that a full understanding of the biology of PDZ proteins will require a comprehensive insight into the intricate relationships between PDZ domains and their peptide and lipid ligands.

## Introduction

Efficient and accurate flow of cellular information relies on scaffolding proteins that coordinate the physical assemblies of signaling complexes [1]. Scaffolding proteins are generally composed of multiple modular interaction domains. PSD-95/DLG1/ZO-1 (PDZ) domains are among the most abundant modular domains in multicellular organisms. In human, there are more than 250 PDZ domains, present in over 150 different proteins that commonly control processes of cell signaling and polarity [2], [3]. PDZ domains consist of 80 to 90 amino acids with a typical fold of six β-strands flanked by two α-helices. The first PDZ domains were identified about twenty years ago [4], [5], [6], and were early on shown to recognize C-terminal ends of target proteins [7], [8]. Later, they were found to also interact with internal peptide stretches and to hetero- or homo- dimerize [9], [10]. More recently, studies from our group revealed that some PDZ domains interact with membrane lipids, in particular phosphoinositides (PtdInsPs) [11], [12], [13].

PtdInsPs control various aspects of cell signaling, vesicular trafficking, and cytoskeleton remodeling, and emerge as important regulators of cell polarization [14], [15]. This makes the PDZ-PtdInsPs connection conspicuous. The differential subcellular compartmentalization of the seven biologically relevant PtdInsPs is crucial for cell function and is regulated by an intricate system of phosphatases and kinases. For example, the plasma membrane is enriched in PtdIns(4,5)P2, while early endosomes and the trans-Golgi network are enriched in PtdIns3P and PtdIns4P, respectively. The nucleus has its own PtdInsPs system, but the exact compartmentalization of nuclear lipids remains a conundrum [16], [17]. The PtdIns phosphorylation code can be read by various protein modules including PH, FYVE, PX, ENTH, CALM, PTB and FERM domains [18]. In the few cases investigated, the PDZ domain-mediated PtdInsPs interactions appear highly relevant for the function of the proteins in question [12], [19], [20].

Until now, few studies have focused on the prevalence, specificity and determinants of PDZ-PtdInsPs binding-interactions. In a first extensive study, Wu et al, baited 74 isolated purified PDZ domains and 14 PDZ tandems with liposomes prepared from bovine brain lipid extracts [21] and estimated that up to 20 percent of the human PDZ domains might interact with membrane lipids [21]. In a complementary study, with 70 PDZ domains from various species, Chen et al showed that up to 40% of PDZ domains interact with plasma membrane mimetic vesicles [22]. For 4 out of 28 PDZ domains tested, they found evidence for selective PtdInsPs binding [22]. Different attempts have been made to identify the phospholipid binding site. NMR analysis of the second PDZ domain of PAR-3 identified a defined cluster of basic residues, situated in proximity of the canonical peptide carboxylate binding site, as a PtdIns3P head group docking site [21]. In contrast, mutagenic analysis of the PDZ domains of PICK1, DegP syntenin-1 and syntenin-2 suggested other lipid interacting regions, overlapping or not with the canonical peptide binding site [13], [23], [24], [25]. Based on modeling approaches, Chen et al. also predicted that lipid and peptide binding sites can overlap or not [22]. Several studies have indicated that peptide and PtdInsPs binding are competitive [13], [21], [22], [26], but a recent study described, for the first time, a case of synergistic peptide and PtdInsPs binding [27], suggesting that PDZ domain localization may be driven by a combination of peptide and lipid recognition.

In this study we aimed to clarify whether and how particular PDZ domains mediate specific PtdInsPs-interaction and whether synergistic PDZ/peptide/PtdInsPs interplay is more than anecdotal. To this end we screened the human PDZ proteome for PtdInsPs interactions combining cell-localization assays and *in vitro* binding experiments. We established the affinities and specificities of these interactions and probed, for two selected cases, the interplay between peptide and PtdInsPs binding. We identify a high pI value and clusters of basic residues as common features of PtdInsPs-interacting PDZ domains and successfully predict additional PtdInsPs binding PDZ domains.

## Results and Discussion

### Identification of Candidate PtdInsPs Binding PDZ Domains from a Cell-based Screen

**Figure 1:**
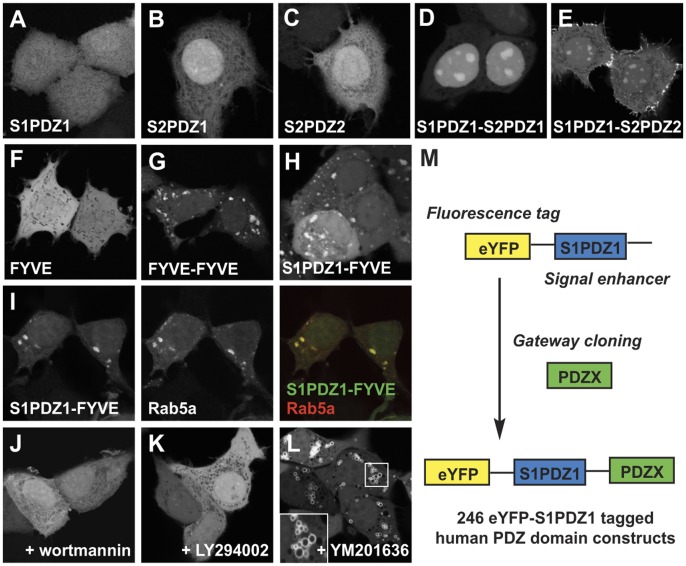
The PDZ1 of syntenin-1 function as an enhancer for PtdInsPs dependent cellular localization. A–L. Confocal micrographs illustrating the subcellular localization of different fluorescent constructs transiently over-expressed in MCF-7 cells. The eYFP-S1PDZ1 (screening construct) localizes diffusely (A). The eYFP-tagged PtdInsPs binding PDZ1 and PDZ2 domain of syntenin-2 show diffuse localization in the cytoplasm and the nucleoplasm when taken in isolation (B,C), but concentrate in subnuclear regions and at the plasma membrane when fused to S1PDZ1 (D, E). Distribution of the tandem repeat of the FYVE domain of Hrs (G), a probe for early endosomal PtIns(3)P. Fusion of eYFP-S1PDZ1 with a single FYVE domain of Hrs, that distributes diffusely when taken in isolation (F), results in the concentration of the fluorescence on vesicular structures (H) that co-localize with the endosomal marker eCFP-Rab5a (I). Treatment with the PtdIns-3-kinase inhibitors wortmannin (J) or LY294002 (K) induces release of the construct from the vesicles. The PtdIns(3)P dependent localization was further confirmed by treatment of the cells with the YM201636 PtdIns(3)P-5-kinase inhibitor inducing swollen PtdIns(3)P-rich vesicles (L). M. Scheme of the cloning strategy.

We used cell-localization as an assay for the identification of candidate PtdInsPs interacting PDZ domains. Briefly, the screening vector encoded a fluorescent tag (enhanced Yellow Fluorescent Protein, eYFP) linked to an ‘enhancer element’ (the first PDZ domain of syntenin-1, S1PDZ1, which by itself binds weakly to PtdInsPs [13]).The syntenin PDZ1-PDZ2 tandem has an apparent affinity of 44 µM for PtdIns(4,5)P2 in the background of liposomes mimicking the composition of the plasma membrane [28]. Transiently over-expressed eYFP-S1PDZ is diffusely localized in MCF-7 cells (Fig. 1A), but the protein becomes targeted to subcellular loci that are enriched in specific PtdInsPs if combined with a PtdInsPs interacting module, as shown for the first and the second PDZ domain of syntenin-2 (S2PDZ1 and S2PDZ2) [12]. When expressed in isolation, the S2PDZ1 and S2PDZ2 domains localize diffusely in the cells (Fig. 1B and C). However, when fused to S1PDZ1 they are enriched in PtdIns(4,5)P2 subcellular compartments, in 84±0.8 and 83±3.3 percent of cells, respectively (Fig. 1D and E). To clarify whether the read-out would work for other subcellular PtdInsPs pools, we challenged the screening system with the FYVE domain of Hrs, known to interact with PtdIns3P at early endosomes, but concentrating on these vesicles only when expressed as a FYVE-FYVE tandem [29] (compare Fig. 1F to Fig. 1G). Transiently over-expressed eYFP-S1PDZ1-FYVE localized to vesicular structures in 72±7.5 percent of MCF-7 cells (Fig. 1H), which co-localized with the early endosomal marker eCFP-Rab5a [30] (Fig. 1I). The endosomal enrichment of eYFP-S1PDZ1-FYVE was lost upon treatment with the PtdIns 3′ kinase inhibitors wortmannin [31] and LY294002 [32] (Fig. 1J and 1K). Additionally, inhibition of PtdIns3P 5′kinase by YM201636, which leads to the formation of PtdIns3P enriched vesicles [33], resulted in accumulation of eYFP-S1PDZ1-FYVE at the limiting membranes of these structures (Fig. 1L). The data thus indicate that the screening approach has the potential to identify domains interacting with different PtdInsPs pools.

**Figure 2:**
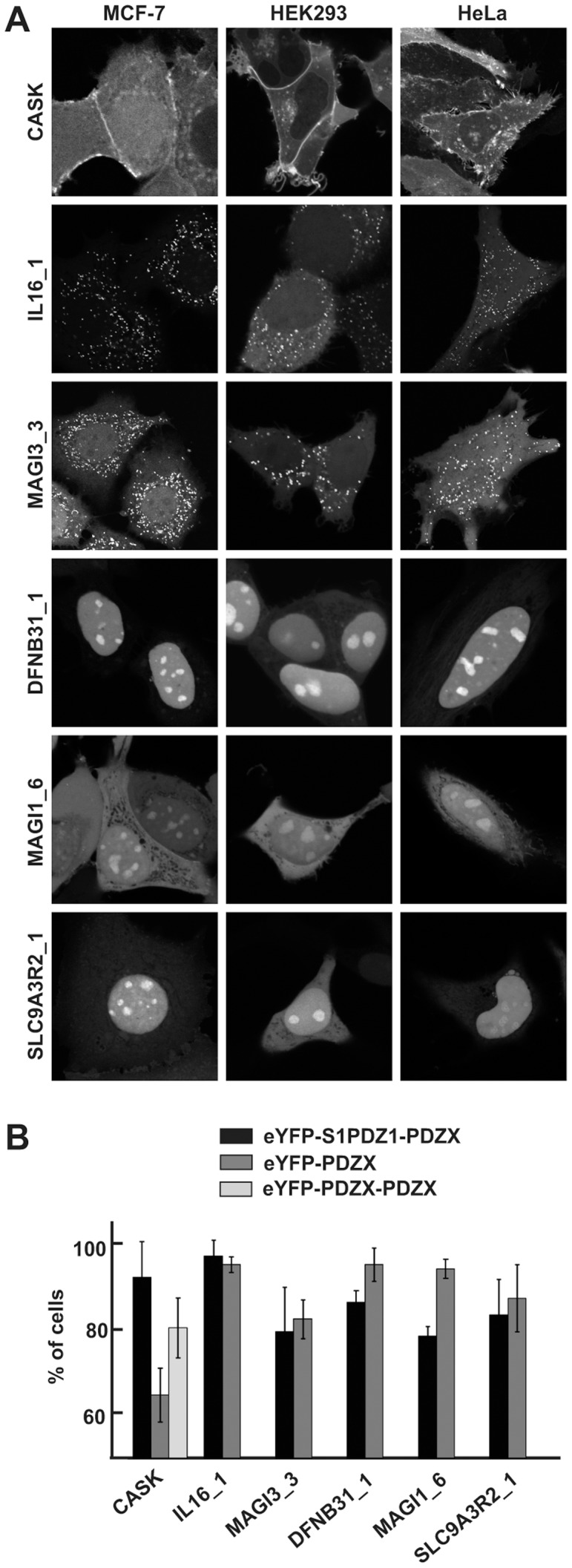
Subcellular distribution of human PDZ domains when fused to eYFP-S1PDZ1. **A.** Confocal micrographs of MCF-7, HEK293 and HeLa cells transiently over-expressing selected eYFP-S1PDZ1 tagged PDZ domains, as indicated. Shown are examples of strong plasma membrane localization (CASK), bright cytosolic spots (MAGI3_3 and IL16_1) and enrichment in subnuclear organelles (DFNB31_1, MAGI1_6 and SLC9A3R2_1). **B.** Bar graph illustrating the mean percentage of MCF-7 cells (± S.D.) where the eYFP-S1PDZ1-tagged and eYFP-tagged PDZ domains were enriched in the characteristic subcellular compartments.

We introduced 246 PDZ domains into the screening vector by Gateway cloning (Fig. 1M). The PDZ domains are named by their host protein gene name (e.g. CASK), or the name followed by an index in case of multiple PDZ domains from the same protein (e.g. MAGI1_1). The constructs were transiently over-expressed in MCF-7 cells. Fluorescence intensities of the overexpressed constructs were similar, and constructs were of the expected size with no evidence for proteolysis (Fig. S1A–B). The intracellular localization was investigated by fluorescence wide-field microscopy, and when needed by confocal microscopy (Fig. 2A, Fig. S2). In the majority of the cases (194), the fluorescence distribution was diffuse. Yet, 53 PDZ domains mediated discrete to distinctive enrichments of the fluorescence at specific locations, potentially reflecting PtdInsPs-rich subcellular compartments. These domains were assigned to one or more of the following categories: I) discrete plasma membrane localization (24 cases), II) strong plasma membrane enrichment (2 cases), III) cytosolic spots (8 cases) and IV) concentration in subnuclear organelles (22 cases) (see Table 1, Fig. 2).

**Table 1:** Distinct distributions of human eYFP-S1 PDZ1-PDZX in MCF-7 cells as determined by wide-field and/or confocal microscopy.

	Protein (eYFP-S1PDZ1-PDZX)
Discrete plasma membrane	APBA1_1, APBA1_2, APBA3_2, DLG1_2, DLG2_2, DLG4_3, HTRA1, LIN7A, LNX2_1, MAGI3_6, MLLT4, MPP1, MPP6, MPP7, PARD3_1, PARD6A, PARD6G, PDZD3_4, PDZK1_1, PDZRN3_1, PDZRN3_2, PPP1R9B, SHANK1, SYNPO2
Strong plasma membrane	CASK, MPDZ_7
Bright cytosolic spots	DEPDC6, IL16_1, LNX1_4, LNX2_1, LNX2_4, MAGI3_3, PDZD7_2, RAPGEF6
Subnuclear organelles	DEPDC2_2, DEPDC6, DFNB31_1, DFNB31_3, GRIP2_6, IL16_3, MAGI1_6, MAGI2_4, MPDZ_6, PARD3_1, PDZD11, PARD6G, SCRIB_4, SDCBP2_1, SDCBP2_2, SIPA1L1, SHANK1, SLC9A3R1_1, SLC9A3R2_1, SLC9A3R2_2, SNTG1, TJP3_1

The out-come of the screen was largely cell-line independent, as shown by comparison of the subcellular localization of 15 selected constructs (amongst the different categories) in MCF-7, HEK293 and HeLa cells (Fig. 2A, Fig. S2). Only eYFP-S1PDZ1-PDZD2_3 displayed cell-line dependent localization. It showed an atypical filamentous localization in MCF-7 cells, but a subnuclear enrichment in HEK293 and HeLa cell lines, which might indicate that the targeting of the protein is dependent on a peptide strictly expressed in MCF-7 cells. Finally, we investigated to what extent the enhancing element S1PDZ1 was crucial for conferring the subcellular enrichments. We compared eYFP-S1PDZ1- to eYFP-tagged PDZ domains from different categories. For MAGI3_3, IL16_1, DFNB31_1, MAGI1_6 and SLC9A3R2_1 no significant difference could be observed. Yet for CASK, S1PDZ1 had a significant enhancing effect on the membrane targeting (Fig. 2B, Fig. S1C). Making a PDZ tandem construct of CASK (eYFP-CASK-CASK) had a similar effect as adding S1PDZ1. As thirty percent of PDZ domains are expected to form dimers [34], we investigated the oligomeric status versus the influence of S1PDZ1. Interestingly, CASK behaves as a monomer and DFNB31_1, SLC9A3R2_1, IL16_1 are dimerizing (Table S1). However, MAGI1_6 and MAGI3_3 are strict monomers and still localize to defined subcellular domains independently of S1PDZ1 (Table S1, Fig. 2B).

### PtdInsPs-dependent Cellular Localizations *versus* PtdInsPs binding *in vitro*

**Figure 3:**
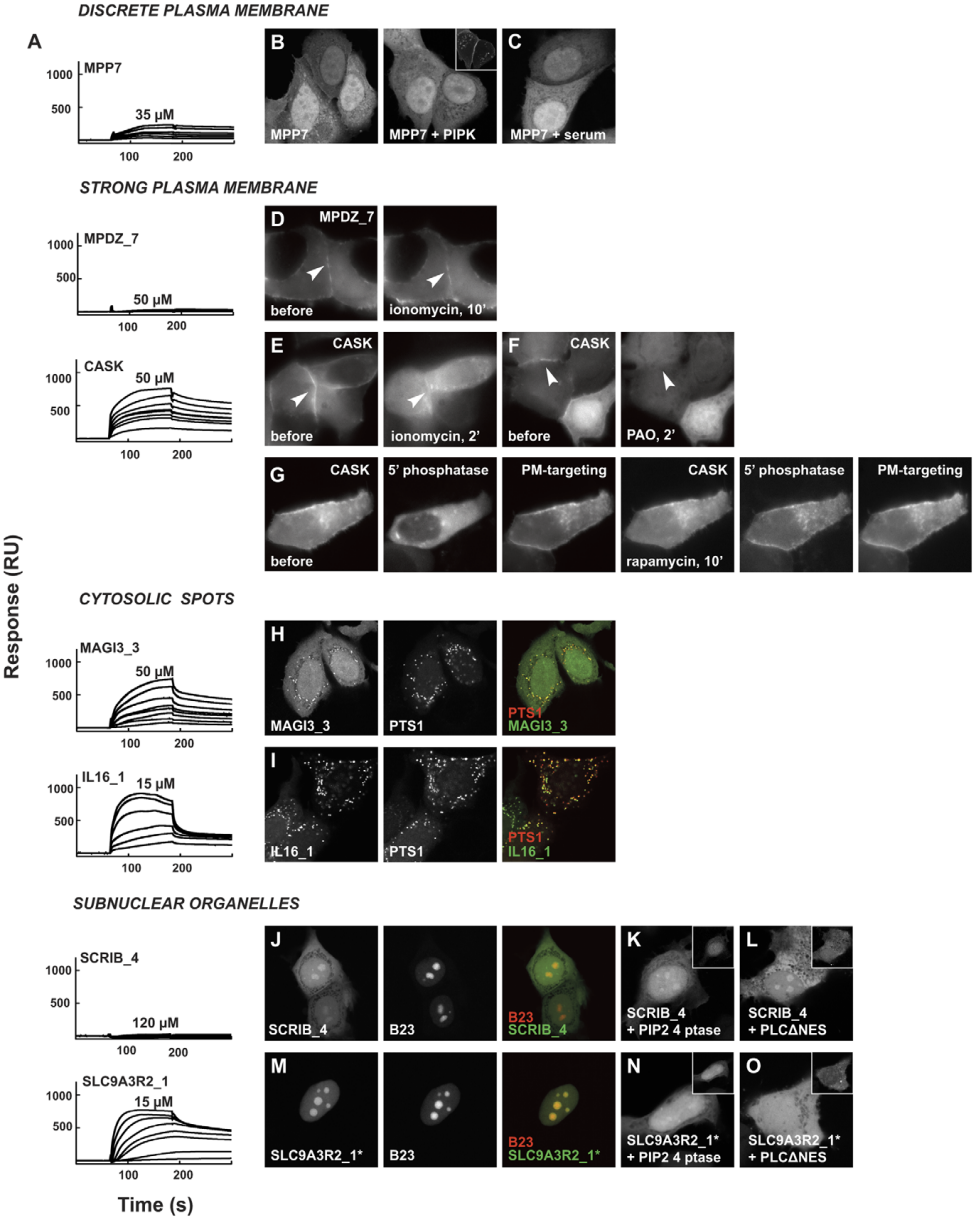
Subcellular distribution of PDZ domains and effects of PtdInsP-modifying treatments compared to *in vitro* PtdIns(4,5)P2-binding. **A.** Double reference subtracted sensorgrams of recombinant his-tagged PDZ domains injected, in a range of concentrations with the highest concentrations used indicated, over 5% PtdIns(4,5)P2 containing DOPC liposomes. **B–O.** Confocal or wide-field micrographs of MCF-7 cells transiently over-expressing selected eYFP-S1PDZ1-tagged PDZ domains (with the exception of SLC9A3R2_1* where S1PDZ1 was omitted). Sensorgrams are provided to the left of the micrographs for each PDZ domain under study. **B**. Co-expression of eYFP-S1PDZ1-MPP7 with PtdIns(4)P-5-kinase (PIPK; shown in the insets) to probe the effect of increased plasma membrane PtdIns(4,5)P2 levels on the distribution of the fluorescent protein. Note the absence of membrane enrichment of the fluorescence upon kinase over-expression. **C**. Serum stimulation of eYFP-S1PDZ1-MPP7 expressing cells to investigate the effect of increased plasma membrane PtdIns(3,4,5)P3 levels. Note the absence of membrane enrichment of the fluorescence upon serum stimulation. For control experiments of B and C, see Fig. S3A–B. **D–G** Ionomycin treatment (D, E), inhibition of PtdIns(4)-kinases by PAO (F) and rapamycin-induced membrane recruitment of PtdInsPs 5′ phosphatase to investigate how reducing PtdIns(4,5)P2 levels affect the membrane enrichment of eYFP-S1PDZ1-MPDZ_7 (D) and eYFP-S1PDZ1-CASK (E-G). **H–I.** Co-expression with mCherry-PTS1 identified the bright cytosolic spots enriched in eYFP-S1PDZ1-MAGI3_3 (H) and eYFP-S1PDZ1-IL16_1 (I) as peroxisomes. Co-expression with mCherry-B23 established that the subnuclear organelles enriched in eYPF-S1PDZ1-SCRIB4 (J) and eYFP-SLC9A3R2_1* (M) corresponded to nucleoli. Co-expression with a PtdIns(4,5)P2-4-phosphatase (PIP2 4 ptase, shown in the insets) or PCLΔNES, did not affect the nucleoli enrichment of eYFP-S1PDZ1-SCRIB4 (K, L) but induced a shift of eYFP-SLC9A3R2_1 (N, O) towards the nucleoplasm and the cytosol.

**Figure 4:**
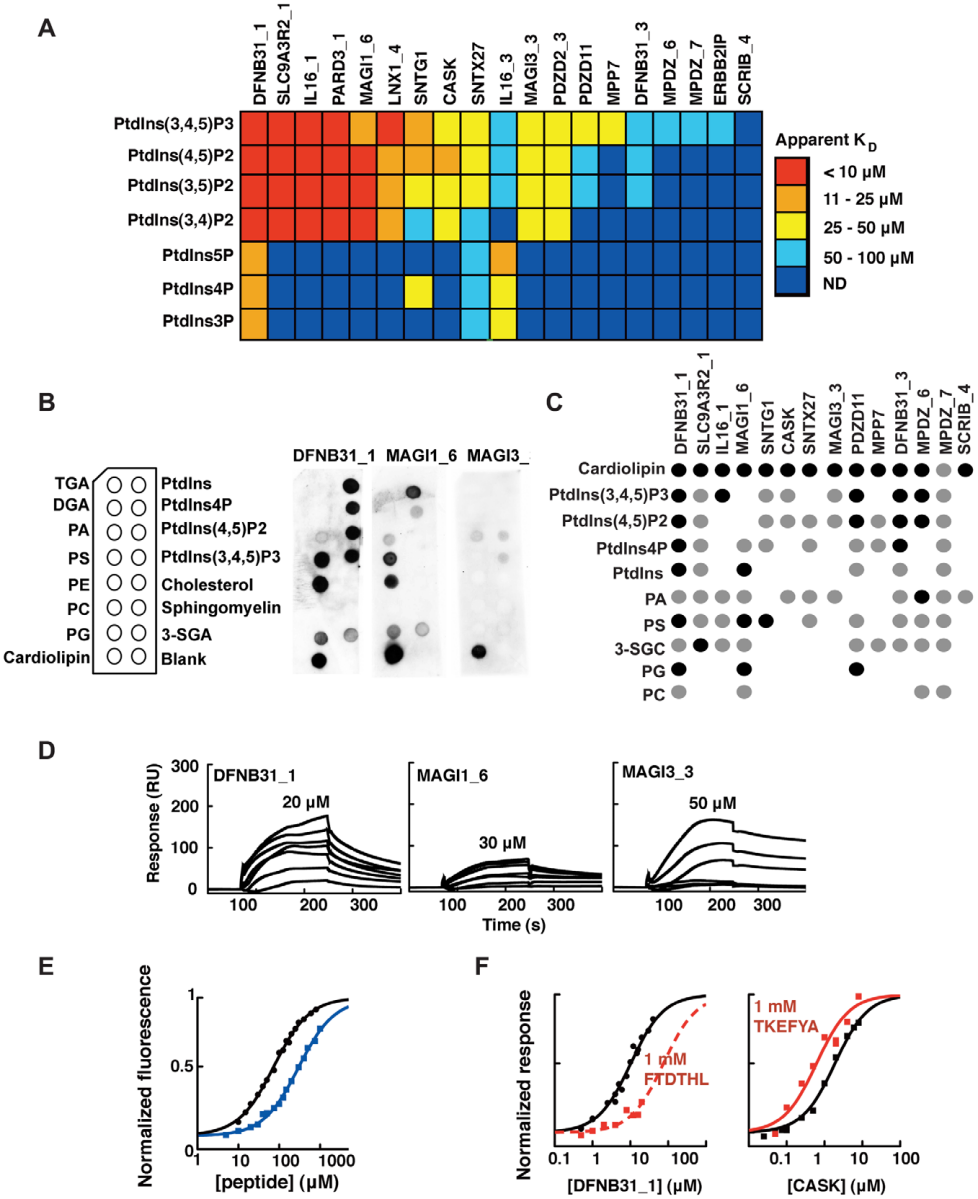
Lipid binding profiles of PDZ domains and differential effects of peptide ligands on PtdInsPs binding. **A**. Apparent PDZ-PtdInsPs affinities as determined by SPR equilibrium binding experiments between his-tagged PDZ domains and 5% PtdInsPs (DOPC liposomes). The color code is indicated to the right. Dark blue color indicates that values were not determinable, for apparent K_D_ values see Table S2. **B**. Schematics of the pre-spotted lipid blot membrane together with representative lipid blots of DFNB31_1, MAGI3_3 and MAGI1_6. **C**. Overview of the lipid blot analysis. Detected spots are represented by black filled circles for intense signals or grey for weaker signals, as compared within each membrane. No binding was detected for TGA, DGA, PC, cholesterol or sphingomyelin. **D.** Double reference subtracted sensorgrams of DFNB31_1, MAGI1_6 and MAGI3_3 injected over 10% PS containing DOPC liposomes. The highest protein concentration used is indicated for each protein. **E.** Equilibrium peptide binding titrations of DFNB31_1/Y167W and the C-terminal peptide of usher (FTDTHL, black circles and black line), and CASK/I517W and the C-terminal peptide of syndecan2 (TKEFYA, blue squares and blue line) as followed by changes in intrinsic tryptophan fluorescence. The protein concentrations were kept constant at 3 µM. **F**. Equilibrium binding isotherms of DNB31_1 (left panel) and CASK (right panel) to 5% PtdIns(4,5)P2 (DOPC liposomes) as determined by SPR experiments in absence (black lines) and presence (red lines) of 1 mM of respective peptide ligands. Note the apparent decrease and increase in affinity in presence of peptide for DFNB31_1 and CASK, respectively.

Defined subcellular localizations of fluorescently tagged PDZ domains may be PtdInsPs and/or peptide driven. For a subset of PDZ domains we therefore investigated to what extent the cellular enrichments were PtdInsPs dependent, and correlated this with PtdInsPs binding *in vitro*. We established the identity of the subcellular compartments by co-localization experiments with known markers, and probed the PtdInsPs dependence of the localizations by treatments altering cellular PtdInsPs levels. The *in vitro* binding was studied by SPR, whereby recombinant PDZ domains were injected over PtdInsPs-containing liposomes. Sensorgrams were corrected for binding to reference (PtdIns-containing) liposomes, and for buffer effects (double reference subtracted), see Fig. 3A for representative sensorgrams. Apparent affinities were determined by equilibrium analysis [35], whereby typically seven different protein concentrations were injected over the immobilized PtdInsPs and the observed equilibrium binding responses were plotted as a function of protein concentrations. Data were fitted by a 1∶1 binding isotherm (GraphPad, Prism) yielding the apparent affinities shown in Fig. 4A and Table S2.

#### Discrete plasma membrane localization

From the twenty-four PDZ domains that localized discretely at the plasma membrane, we selected eight (DLG4_3, LIN7A, MPP7, PARD3_1, PARD6A, PARD6G, PDLIM5, PDZK1_1) and co-transfected them with PtdIns4P5-kinase (PIP5K), which increases the plasma membrane PtdIns(4,5)P2 levels [36]. We were confident of the effectiveness of our PIP5K expression vector as it induced an increased plasma membrane enrichment and a decreased cytoplasmic signal of eYFP- tagged PH domain of PLC, a well-established probe for PtdIns(4,5)P2 [37]. Also the number of cells where eYFP-PH-PLC concentrated at the plasma membrane increased from 84% to 95% (p value = 0.003). Additionally, intracellular spots of overexpressed PIP5K co-localized with eYFP-PH-PLC (Fig. S3A). Similar results were obtained for the PDZ tandem of syntenin-1 (data not shown). On the contrary, none of the eYFP-S1PDZ1-tagged PDZ domains was affected by PIP5K overexpression, arguing against a PtdIns(4,5)P2-dependent membrane targeting of this category of PDZ domains (Fig. 3B, Fig. S3C).

We performed SPR binding experiments with MPP7 PDZ, randomly selected from this group, testing its binding to various PtdInsPs species. The binding responses were low in the protein concentration range used (up to 50 µM with the limit being set by the solubility of the protein), except for PtdIns(3,4,5)P3 (25±8 µM) (Fig. 4A, Table S2), suggesting mostly low affinity interactions. The *in vitro* binding data thus suggested a potential contribution of PtdIns(3,4,5)P3 in membrane targeting of MPP7, but we failed to demonstrate such contribution *in vivo.* Transient increase in plasma membrane PtdIns(3,4,5)P3 levels by serum stimulation did not promote increased enrichment of eYFP-S1PDZ1-MPP7 (Fig. 3C) while it had a clear effect on the plasma membrane enrichment of the eGFP-tagged PH domain of Akt, a well-established probe for PtdIns(3,4,5)P3 [37] (Fig. S3B). Similarly, serum stimulation had no effect on the cellular targeting of the other seven PDZ domains investigated (DLG4_3, LIN7A, PARD3_1, PARD6A, PARD6G, PDLIM5, PDZK1_1) (Fig. S3C). We therefore concluded that the PDZ domains that we investigated in this category most probably do not rely on PtdInsPs for their discrete membrane localization.

#### Strong plasma membrane enrichment

Two constructs, eYFP-S1PDZ1-CASK, and eYFP-S1PDZ1-MPDZ_7 were strongly enriched at the plasma membrane. The PtdInsPs dependence of the enrichment of these two constructs was probed by ionomycin treatment, which induces breakdown of plasma membrane PtdIns(4,5)P2 [38]. Ionomycin treatment caused a loss of membrane localization of eYFP-S1PDZ1-CASK (Fig. 3E), but not of eYFP-S1PDZ1-MPDZ_7 (Fig. 3D), suggesting a PtdIns(4,5)P2-dependent membrane localization of the former, but not the latter fusion protein. Corroborating a role for PtdInsPs in the plasma membrane targeting of CASK, treatment with phenylarsine oxide (PAO), which inhibits the PtdIns 4′kinases [39], [40], released eYFP-S1PDZ1-CASK from the plasma membrane (Fig. 3F) and eYFP-S1PDZ1-CASK concentrates around Arf6Q67L macropinosomes enriched in PtdIns(4,5)P2 [27] (data not shown). However, the rapamycin inducible translocation system of a PtdInsPs 5′ phosphatase developed by Varnai *et al.* [41], which induces a decrease of plasma membrane PtdIns(4,5)P2, failed to delocalize eYFP-S1PDZ1-CASK from the plasma membrane (Fig. 3G). PIP5K overexpression also failed to increase the plasma membrane recruitment of eYFP-CASK, but intriguingly overexpression of a dominant negative form of PIP5K lacking half of its kinase domain caused plasma membrane enrichment of eYFP-CASK (data not shown). Treatment of cells with dibucaine, or wortmannin failed to release eYFP-S1PDZ1-CASK from the plasma membrane, excluding respectively a major role for PS and PtdIns 3′kinase products in the targeting (data not shown). The above treatments were also performed for the eYFP-CASK-CASK construct, with similar outcome (Fig. S3D-F). SPR experiments established that MPDZ_7 has low affinity for PtdInsPs (apparent K_D_ generally above 100 µM). CASK interacts with monophosphate PtdInsPs with low affinity (>100 µM K_D_) but has good affinity for polyphosphate PtdInsPs, with an apparent affinity of 25±7 µM for PtdIns(4,5)P2 (Fig. 4A, Table S2). MPDZ_7 is thus most likely targeted to the plasma membrane by protein-protein interactions. In contrast, the experiments suggest that PtdInsPs are novel physiological regulators of CASK. While we would favor an important role for PtdIns(4,5)P2 because of its abundance at the plasma membrane, the precise PtdInsPs metabolizing enzymes controlling the presence of CASK at the plasma membrane require deeper investigation.

#### Cytosolic spots

Five out of the eight eYFP-S1PDZ1-PDZX constructs enriched on cytosolic spots, suggestive of endomembranes, were investigated by live cell fluorescence microscopy. The LNX1_4, LNX2_1 and PDZD7_2 spots corresponded to inert structures that most probably resulted from protein aggregation and we excluded them from further microscopy experiments. In contrast, the cytosolic spots visible upon over-expression of eYFP-S1PDZ1-MAGI3_3 and eYFP-S1PDZ1-IL16_1 corresponded to dynamic vesicles. These fluorescent proteins did not co-localize with established markers of early or late endosomes (EEA1 and CD63 respectively [42], [43], data not shown), but with the peroxisomal markers PTS1 (Fig. 3H and I) and pex14p (not shown) [44], [45]. Peroxisomes have a unique PtdInsPs system [46], but there is no established treatment for modulating their PtdInsPs levels. The treatments we attempted, wortmannin and LY294002, affecting PtdIns3P levels, and ionomycin, reducing PtdIns(4,5)P2 levels, did not alter the peroxisomal enrichments of the fluorescent proteins. Yet recombinant IL16_1 and MAGI3_3 interacted with various PtdInsPs *in vitro*, with high-to-intermediate affinities (Fig. 4A, Table S2). PDZ domains might thus bind peroxisomal PtdInsPs. Identifying these PtdInsPs and the functional relevance of these interactions constitute a potential innovative line of research.

#### Subnuclear organelles

Twenty-two proteins were enriched in subnuclear organelles, some corresponding to nucleoli as shown by co-localizations of SCRIB_4 and SLC9A3R2_1 with the nucleolar marker nucleophosmin/B23 [47] (Fig. 3 J and M).

**Figure 5:**
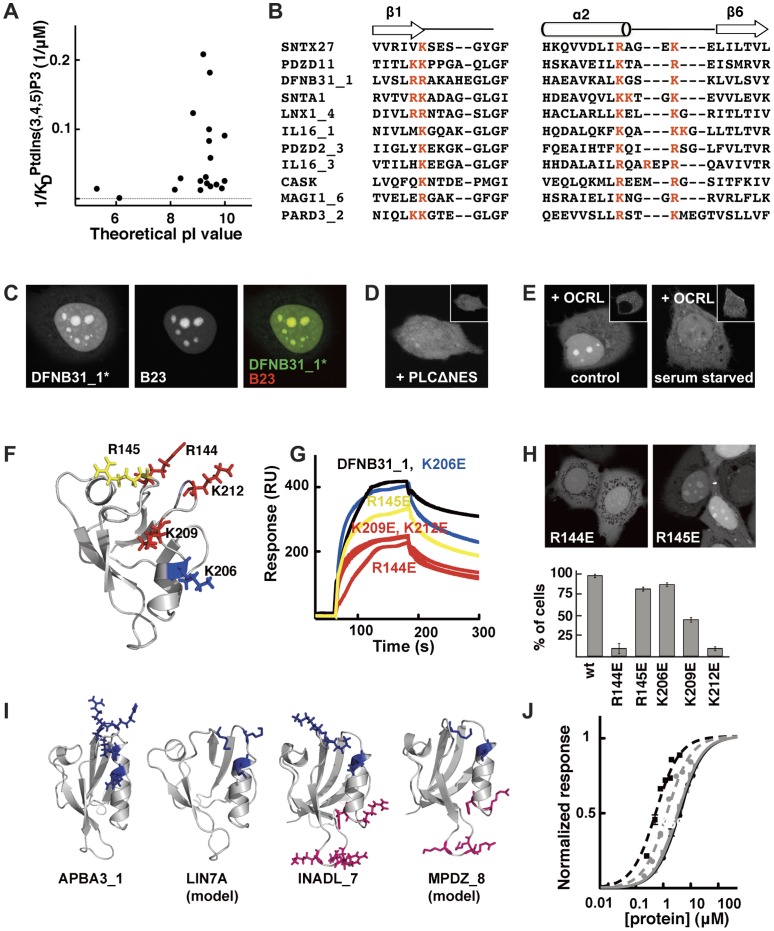
Common features of PtdInsPs interacting PDZ domains. **A.** Apparent affinities of individual PDZ domains for PtdIns(3,4,5)P3 (Table S2) plotted *versus* their theoretical pI values. **B.** Partial sequence alignment highlighting the conserved positive charge cluster (red letters) shared by eleven PtdInsPs-interacting PDZ domains. **C**. Confocal micrographs showing the co-localization of eYFP-DFNB31_1* (absence of the S1PDZ1 enhancer) with the nucleolar marker B23. Nucleolar enrichment of eYFP-DFNB31_1* relies on PtdInsPs as co-expression with mCherry-PLCΔNES (**D**) or serum mediated translocation of mCherry-OCRL (**E**) shifts expression of eYFP-DFNB31_1* towards the nucleoplasm and the cytoplasm. **F.** Mutagenic analysis of DFNB31_1. Residues probed by site-directed mutagenesis are indicated in the structure (1UEZ) and were colored according to their effects on PtdIns(4,5)P2 binding: blue, no significant effect; yellow, intermediate effect; red, strong effect. **G.** Representative double reference subtracted sensorgrams of wild-type and mutant DFNB31_1 proteins (4 µM) injected over 5% PtdIns(4,5)P2-containing DOPC liposomes. The effects of the mutations on the nucleolar enrichment of DFNB31_1 are shown in **H**. The bar graph illustrates the percentage of transfected cells where strong nucleolar enrichment was observed in confocal microscopy. **I.** Structures of PDZ domains (APBA31_1, 2YT7; LIN7A, swiss model; INADL_7, 2DAZ; and MPDZ_8, swiss model) having theoretical pI values higher than 7 and defined positive charge clusters (blue and pink stick representations) suggesting potential PtdInsPs-interaction. **J.** Normalized equilibrium binding isotherms of APBA31_1 (black squares and black dotted line), LIN7A (grey circles and grey dotted line), INADL_7 (black circles and black line) and MPDZ_8 (grey squares and grey line) to 5% PtdIns(4,5)P2 in DOPC liposomes as determined by SPR experiments.

A limited literature suggests the presence of PtdInsPs in nucleoli [12], [48]. We investigated the potential PtdInsPs dependence of the nucleolar enrichments for six randomly selected fusion proteins (DFNB31_1, DFNB31_3, MPDZ_6, SCRIB_4, SNTG1 and SLC9A3R2_1). eYFP-SLC9A3R2_1 responded to alterations of the cellular PtdInsPs levels: it was shifted from the nucleoli towards nucleo- and cytoplasm upon co-expression with the *Shigella* PtdIns(4,5)P2 4′ phosphatase IpgD (Fig. 3N), as well as upon co-expression with yeast phospholipase C1 deleted for its Nuclear Export Signal (PLCΔNES) (Fig. 3O). In contrast, eYFP-S1PDZ1-SCRIB_4 was insensitive to the lipid-modifying treatments (Fig. 3K and L). The data thus suggest that targeting to nucleoli of SLC9A3R2_1, but not SCRIB_4 is PtdIns(4,5)P2 dependent. In line with the *in vivo* data, recombinant SLC9A3R2_1 interacted with high affinities with different PtdInsPs species *in vitro* (Fig. 4A, Table S2) while SCRIB_4 did not interact with any PtdInsPs species. Similarly, the outcome of lipid modifying treatments corresponded well with *in vitro* PtdInsPs binding properties of the four other investigated domains. DFNB31_1 and SNTG1 showed high-affinities for PtdInsPs *in vitro* and were sensitive to PtdInsPs modifying treatments, while DFNB31_3 and MPDZ_6 were not (Fig. 4A, Table S2, Fig. S3G, Fig. 5 D-E). Two previous studies showed that the PDZ proteins syntenin-2 (SDCBP2) and zonulin-2 control the enrichment of PtdIns(4,5)P2 in nuclear speckles, interchromatic splicing and transcription factories [12], [26]. The high number of PDZ domains localizing to subnuclear organelles is intriguing. It would be interesting to further investigate whether cross-talk with nuclear PtdInsPs is a common theme in the biology of PDZ proteins and what the functional consequences are of such interactions.

### Specificity of PtdInsPs-interacting PDZ Domains

For a more comprehensive view of PDZ-PtdInsPs binding affinities and specificities, we investigated the *in* *vitro* PtdInsPs binding of 19 PDZ domains produced as isolated his-tagged recombinant domains. We used PDZ domains belonging to the different subcellular localization categories, and also included two diffusely localized proteins (ERBP2IP and SNTX27) (Fig. 4A and Table S2). ERBP2IP was chosen at random, and SNTX27 was selected as it had previously been suggested as a phospholipid-binder by Pan *et al.* [25]. The apparent PDZ-PtdInsPs affinities ranged from low-to-mid micromolar (>10 µM to <100 µM), with a trend of higher affinities for more phosphorylated species. Among the ten best PtdInsPs-binders solely four behave as strict monomers (Table S1) suggesting that multimerization favor PtdInsPs-interaction. The rather low head-group specificity is in line with studies on other PtdInsPs binding modules, such as several PH domains [49]. Of the two PDZ domains selected for their diffuse cellular localization, ERBPI2P displayed very low affinity for PtdInsPs (>100 µM, except for PtdIns(3,4,5)P3 ∼70 µM), while SNTX27 interacted with PtdInsPs with K_D_ values of on average 50 µM, showing that our cell-localization screen might have missed PDZ domains interacting with PtdInsPs with such modest affinity.

As an alternative *in vitro* approach we performed lipid blot assays using commercially available membranes pre-spotted with PtdIns, PtdIns4P, PtdIns(4,5)P2 and PtdIns(3,4,5)P3 as well as other abundant lipids (Fig. 4B–C). IL16_3 and PDZD2_3, were excluded from this analysis due to their high binding to the blank immobilized spot. ERBB2IP, LNX1_4 and PARD3_1 did not produce any detectable signals. Other PtdInsPs-PDZ interactions were confirmed. Discrepancy between SPR and lipid blot approaches can be easily explained by the fact that the latter method is highly k_off_-dependent. The lipid blot analysis also suggested interactions with other, predominantly anionic, lipids such as cardiolipin, phosphatidic acid (PA) and phosphatidylserine (PS), but not with zwitter ionic lipids such as triacylglycerol (TGA), diacylglycerol (DGA), phosphatidylglycerol (PG), sphingomyelin or cholesterol. We therefore performed SPR experiments with five selected PDZ domains (CASK, DFNB31_1, MAGI1_6, MAGI3_3 and SLC9A3R2_1) and two selected lipids, PS and PE, embedded in DOPC liposomes (10% PS or 40% PE in a DOPC using 100% DOPC as reference). Although we did not observe any detectable PDZ-PE binding (data not shown), we detected low affinity interactions with PS (selected sensorgrams shown in Fig. 4D). We have previously reported that weak electrostatic interactions with PS reinforce the interactions of the second PDZ domain of Polychaetoid with PtdIns(4,5)P2 containing liposomes [27]. We therefore investigated, for the same five PDZ domains, how the apparent PtdIns(4,5)P2 affinities were affected by presenting the lipid in the background of liposomes mimicking biological membrane lipid composition (30% PC, 20% PS, 40% PE and 5% PtdIns(4,5)P2). On average, the apparent PDZ-PtdIns(4,5)P2 affinities were 2.5 times higher in the background of composite liposomes (K_D_^PtdIns(4,5)P2^ in composite liposomes: CASK, 9±2 µM; DFNB31_1 2.7±1 µM; MAGI1_6 4.5±1 µM; MAGI3_3 14±2 µM; SLC9A3R2_1 2.5±1 µM). The results confirm that a subgroup of PDZ domains interact with PtdInsPs and suggest that these interactions may be enhanced *in vivo* by interactions with other anionic phospholipids.

### Interplay between PtdInsPs and Peptide Binding

Given the overall low PtdInsPs specificities, and often modest PtdInsPs affinities, it is unlikely that PtdInsPs bindings alone target the PDZ domains to their defined subcellular compartments. Plausibly, peptide interactions are also involved in controlling the subcellular targeting. To address the interplay between PtdInsPs and peptide-binding we chose DFNB31_1 and CASK as models, taking two of the PDZ domains with well-defined cognate peptides. Cognate peptide ligands were the C-terminal peptides of usher (FTDTHL) for DFNB31_1 [50] and the TKEFYA peptide of syndecan-2 for CASK [51]. We measured the PDZ-peptide affinities by a fluorescence-based approach (Fig. 4E), by engineering Trp into the PDZ scaffolds and following the interactions by changes in intrinsic fluorescence, a strategy previously established for other PDZ domains (K_D_-values: DFNB31_1/Y167W for FTDTHL 46±6 µM; CASK/I157W for TKEFYA 280±20 µM) [27], [52]. We then determined the apparent PtdIns(4,5)P2 affinities in presence of a (close to) saturating concentration of respective peptide (1 mM). The FTDTHL peptide appeared to compete for DFNB31_1-PtdIns(4,5)P2 interactions (K_D_^PtdIns(4,5)P2^ in presence of peptide >30 µM, Fig. 4F), which might indicate overlapping binding sites. In contrast, the TKEFYA peptide had a synergistic effect on CASK-PtdIns(4,5)P2 interactions, conferring a 4-fold increase in apparent affinity (K_D_^PtdIns(4,5)P2^ in presence of 1 mM TKEFYA 6.2±2 µM, Fig. 4F). We have previously reported on competitive binding by peptide and PtdIns(4,5)P2 with CASK [13]. However, in these experiments the peptide was linked to sepharose beads and PtdIns(4,5)P2 was present on micelles, which made it more difficult for the PDZ domain to simultaneously interact with the two ligands.

Thus, PDZ-peptide-PtdInsPs interactions can be competitive as well as synergistic depending on the combination of ligands, a topic that deserves systematic future investigations.

### Common Features of PtdInsPs Interacting PDZ Domains

The most striking common property of the PtdInsPs binding PDZ domains is a high pI value, on average higher than 9 (Fig. 5A) as compared to an average pI of 7 for the human PDZ domains. However, a high pI value alone does not provide high affinity for PtdInsPs. Notably, MPDZ_6 and MPDZ_7 are basic molecules but have low affinities for PtdInsPs (Fig. 4A). To identify potential consensus sequence motif(s) we made a structure based sequence alignment of fourteen of the PtdInsPs-binders of this study, together with previously known PtdInsPs-interacting PDZ domains (i.e. PICK1, the PDZ domains of the syntenins, PARD3_2 and SNTA1, Fig. S4). There was no general consensus sequence for PtdInsPs binding PDZ domains, but a subgroup of 11 out of 22 domains shared a basic cluster of three or four Arg or Lys in the vicinity of the carboxylate binding site (Fig. 5B), previously suggested as a PtdIns3P head group docking site for PARD3_2 [21].

To assess the functional importance of the conserved positive charge cluster, we performed a mutagenic analysis of DFNB31_1. *In vivo,* DFNB31_1 is enriched in nucleoli as shown by the co-localization with the nucleolar marker nucleophosmin/B23 (Fig. 5C). DFNB31_1 interacts with high affinities with different PtdInsPs species *in vitro* (Fig. 4A, Table S2) and its nucleolar localization appears to be PtdInsPs dependent as overexpression of mCherry-PLCΔNES shifts the localization of eYFP-DFNB31_1 towards nucleo- and cytoplasm (Fig. 5D). Similar effect are observed upon overexpression of the inositol polyphosphate 5′ phosphatase OCRL (Fig. 5E) that has a strong preference for PtdIns(4,5)P2 as a substrate [53], [54] and that translocates to the nucleus upon serum starvation (Fig. 5E, compare insets). Four Arg/Lys contributing to the basic cluster of DFNB31_1 were replaced with Glu (R144E, R145E, K209E and K212E), as well as a Lys in the vicinity of this region (K206E) (Fig. 5F). We established that the mutations did not alter the stability of the proteins by introducing the same mutations in the background of DFNB31_1/Y167W and determining the urea-induced unfolding of the proteins as followed by fluorescence (Table 2). We determined the effects of the mutations on the PtdIns(4,5)P2 and peptide binding *in vitro* (Fig. 5F–G, Table 2) and on the *in vivo* localization (Fig. 5H). Three mutations, R144E, R209E and K212E, conferred significant losses of PtdIns(4,5)P2 affinities (Table 2), affected the peptide binding (the R144E to less extent) and conferred drastic decreases in nucleolar enrichments *in vivo* (Fig. 5H). The R145E mutation caused a minor, 2-fold decrease in PtdIns(4,5)P2 binding, had similar effects on the peptide binding as R144E but did not affect the cellular localization of the fluorescent protein. The K206E mutation did not affect the *in vitro* PtdIns(4,5)P2 binding, while conferring a 2-fold decrease in peptide binding affinity without shifting the cellular enrichment. Taken together, the data confirm the importance of the conserved basic cluster in PtdInsPs binding and the importance of this charge cluster in defining the cellular localization of the fluorescently tagged protein.However, from the lack of complete conservation of the identified positive charge cluster it is clear that PDZ domains may interact with negatively charged PtdInsPs through alternative ways. Notably, SLC9A3R2_1, is among the highest affinity PtdInsPs binders, but has only two basic residues of the consensus positive charge cluster. Instead, this protein has a basic cluster in the loop region between beta strand 2 and 3 (K29, R31, R32), which might be involved in PtdInsPs binding of this protein.

**Table 2:** Binding characteristics and stability data of DFNB31_1 wild type and mutants.

	*K_D_^app^* PtdIns(4,5)P2 (*µM*)	*K_D_* * FTDTHL (*µM*)	*ΔΔG_D-N_* * *(kcal/mol)*
DFNB31_1	7.8±2	46±6	–
DFNB31_1/R144E	>20	82±3	−0.65±0.05
DFNB31_1/R145E	15±5	71±8	−0.53±0.05
DFNB31_1/K206E	8.2±2	111±8	0.41±0.03
DFNB31_1/K209E	>20	175±15	−0.15±0.01
DFNB31_1/K212E	>20	220±40	1.2±0.1

* A Y167W mutation was introduced in DFNB31_1 and mutants thereof to function as a fluorescent probe.

To validate that a high pI (>7) and a cluster of basic residues could be a signature for PtdInsPs binders we selected four PDZ domains fulfilling the criteria that did not show-up in our screen, and tested their *in vitro* PtdInsPs binding. We chose two PDZ domains with positive charge clusters similar to the main consensus (APBA3_1 and LIN7A) and two domains, INADL_7, and MPDZ_8, that share a similar basic cluster with SLC9A3R_1 (Fig. 5I and Fig. S4). We found that the four PDZ domains interact with PtdIns(4,5)P2 with high affinities (apparent K_D_ values 0.5±0.1 µM, 1.4±0.2 µM, 3.6±0.7 µM and 4.0±0.8 µM for APBA3_1, LIN7A, INADL_7 and MPDZ_8, respectively, Fig. 5J). The results suggest that subgroups of PDZ domains may engage clusters of basic residues structurally located in vicinity of either end of the helix 2 for their interactions with PtdInsPs. Interestingly, the study by Cheng et al [22], published during the revision of this work, reached a similar conclusion. The lack of a unique signature suggests that PtdInsPs bindings have appeared independently in different PDZ domains, which is reminiscent of what has previously been proposed for PH domains [55].

### Concluding Remarks and Discussion

Through a cell-based screen and complementary *in vitro* binding experiments we identified several new PtdInsPs binding PDZ domains and, using various lipid modifying treatments, extensively documented for some of them PtdInsPs-dependent subcellular targeting. Interestingly, we observed PtdInsPs-dependent targeting to the plasma membrane (CASK) as well as to the nucleus (DFNB31_1, SLC9A3R2_1, SNTG1). Our study is the so far most comprehensive cell-based screen for PtdInsPs interacting PDZ domains. It shows that PDZ-PtdInsPs interactions commonly are in the low-to-mid micromolar range and tend to be reinforced by additional electrostatic interactions with other anionic phospholipids such as PS. Binding to cognate peptide ligands can either reinforce or compete with PDZ-lipid interactions, and some PDZ domains are likely to coincidently bind protein and lipid ligands. We established that high pI values and clusters of basic residues are common properties of PtdInsPs interacting domains, a ‘signature’ that can be used to predict additional PtdInsPs-interacting domains. It is thus now clear that a subgroup of PDZ domains interact with PtdInsPs through positively charged clusters, which should be taken into consideration when addressing the biology of PDZ containing proteins.

In the current study we used isolated PDZ domains, which may have affected the outcome in several ways. Indeed, it is not always obvious how to define the borders of PDZ domains and changing the borders may alter the functional properties of the domains [2]. Domain extension may affect the domain functionalities in several different ways such as altering the dynamic properties of the PDZ domains or extending the target ligand-binding pocket as reviewed extensively by Wang *et al* [56]. Of particular interest for this study, we recently reported that basic residues in the C-terminal extension of the PDZ-tandem of syntenin-1 contribute with electrostatic charges to membrane localization [28]. It could hence be interesting to systematically investigate if there is a correlation between PtdInsPs interacting PDZ domains and positively charged domain extensions.

Of further note is that PDZ domains often make part of multi-domain proteins and thus may only provide a part of the peptide and/or lipid interactions required for targeting their host proteins to the appropriate locations. Indeed, PtdInsPs interacting PDZ domains tend to occur in tandem with other PtdInsPs binding modules, such as the PDZ domain of PICK1 being linked to a BAR domain [25] and the lipid binding PDZ domain of SNTA1 being connected to a PH domain [57]. Combinations of more than one lipid binding module provide proteins with avidity for the lipid membrane. Through our experimental design, we tried to compensate for such effects by including S1PDZ1 as a part of the screening constructs. However, it is clear that the design did not completely compensate for such avidity effects as, for example, both PICK1 and SNTA1 were found to be diffusely localized in our cell based screen.

A comparison of the cellular enrichments observed for the PDZ domains in the screening constructs with the reported localizations of the host proteins based on Gene Ontology Annotations (Table S3) reveals a correlation between the two in approximately 60 percent of the cases. This is a reasonable out-come given that our data are derived from over-expression experiments, that the full-length proteins commonly contain multiple interaction domains and that PDZ interactions may be masked in the full-length proteins by autoinhibition [58]. We here provide evidence for domains that are capable of interacting with PtdInsPs, but future experiments are needed to address the potential implications of these interactions on the biological function of the full-length proteins.

Our screen greatly expanded the set of known PtdInsPs interacting PDZ domains and provided signatures that can be used for the identification of additional lipid binding PDZ domains. The PDZ domains we identified as PtdInsPs binders interact with lipid metabolizing enzymes such as PTEN (MAGI3, PARD3, SLC9A3R2), are parts of proteins involved in PtdInsPs dependent processes such as vesicular trafficking (APBA3), interact with nuclear-PtdInsPs or even potentially peroxisomal-PtdInsPs which paves the way for future original research on poorly investigated areas of cell biology.

## Materials and Methods

### Molecular Biology

Our human PDZ domain collection comprises 246 PDZ domains. The domains and their boundaries were established by cross searching Interpro (V18, http://www.ebi.ac.uk/interpro/), PFAM (V23, http://pfam.sanger.ac.uk/) and SMART version 5.0 (http://smart.embl-heidelberg.de<http://smart.embl-heidelberg.de/). Ten amino acids from the original protein sequence were added on each side of the predicted PDZ domains. Predicted amino acid sequence and actual translation of cloned DNA constructs are given in Table S4. Primers, containing Gateway B1 and B2 recombination tails, were designed using the OSP program as described previously [59] including a stop codon before the B2 tail. DNA fragments encoding each PDZ domain were amplified by PCR and cloned into the pDONRZeo Entry vector using the Gateway recombinational cloning system (Invitrogen). PDZ Entry clones were sequence verified using PDONZeo M13 forward and reverse primers and then used in a Gateway LR recombination reaction to transfer the DNA coding for the PDZ domain into the eYFP-S1PDZ1 vector [27] adapted for Gateway cloning by insertion of Gateway cassette into the unique SmaI site of the polylinker. Fusion of PDZ into the expression vector after LR reaction was confirmed by sequencing with the upstream primer: 5′-GATCACATGGTCCTGCTG.

PDZ domains were subcloned from the screening constructs into pEYFP-C2 (Invitrogen) for microscopy assays or into the pETM-11 (EMBL Heidelberg) vector for his-tagged protein expression. Coding sequences were amplified using oligonucleotides carrying restriction sites for *EcoRI* and *SalI*, or *EcoRI* and *NcoI*. The PCR products were digested and ligated into the equally digested peYFP-C2 or pETM-11 plasmids. Site-directed mutagenesis was performed using the Quickchange protocol (Stratagene). The PLCΔNES was described earlier [12], and OCRL was subcloned from a home-made cDNA library from MCF-7 cells. All constructs were verified by DNA sequencing.

### Cell Culture, Transfections and Microscopic Analysis

MCF-7 cells obtained from American Type Culture Collection (Manassas, VA) were cultured in DMEM/F-12 medium (Life Technologies) supplemented with 10% fetal bovine serum (Gibco). For microscopy experiments cells were plated on eight-well chamber slides (Nalge Nunc International, Roche), transfected after 4 h with FuGENE (Roche Diagnostics) and fixed with 4% parafolmaldehyde the day after transfection. Fluorescence confocal micrographs were obtained with Olympus Fluoview 1000 (Olympus) and wide field micrographs with Leica AS-MDW (Leica) using appropriate filter sets. The enrichment of eYFP-S1PDZ1-PDZX, eYFP-PDZX and eYFP-PDZX-PDZX constructs in the defined subcellular regions (plasma membrane, peroxisomes and nucleoli) was scored by confocal microscopy and images of at least 30 cells from three independent experiments were analyzed for each condition. For mutagenic analysis, nucleolar enrichment of eYFP-DFNB31_1 and its mutants was scored in living cells and quantified by measuring fluorescence intensities of nucleoli and nucleoplasm and calculating the nucleolar/nucleoplasm ration after background correction. The ratio was calculated for at least 30 cells from three independent experiments and cells were classified as displaying strong nuclear enrichment if the nucleolar/nucleoplasm ration was higher than 1.2.

### Pharmacological Treatments

Wortmannin (Sigma-Aldrich) or LY294002 (Calbiochem) were added to the cells for 30 min to the final concentration of 500 nM and 50 µM, respectively. Cells were treated with 800 nM of YM201636 (Chemdea) for 2 h. For ionomycin treatment cells were washed with Krebs-Ringer buffer (Varnai et. al., 1998) before addition of 10 µM ionomycin (Calbiochem) in the same buffer. Phenylarsine oxide (PAO) (Sigma-Aldrich) was added to the cells to the final concentration of 100 µM and rapamycin (Sigma-Aldrich) to 200 nM. For serum stimulation experiments cells were serum starved for 18 h before 30 minutes stimulation with 10% fetal bovine serum (Gibco).

### Immunofluorescence

Cells were stained as described previously [58] using anti-Myc antibody (9E10, Sigma-Aldrich) for detection of Myc-tagged PtdInsPs 5′ kinase and anti-pex14p antibody (generous gift from Marc Fransen, Leuven) for visualization of peroxisomes, followed by Alexa594-conjugated goat anti-mouse secondary antibodies (Invitrogen).

### Protein Expressions

N-terminally his-tagged proteins were expressed, in *E. coli* ER2566 cells carrying the pETM-11 expression constructs, and purified by purified by nickel affinity chromatography as described previously [27].

### SPR Experiments

SPR experiments were performed in a BiacoreT100 (GE Healthcare) at 25°C in 25 mM HEPES, 150 mM NaCl, pH 7.2 and 1 mM beta-mercaptoethanol with a flow rate of 30 µl/min. Liposomes were prepared and immobilized on L1 sensor chips as described previously [12]. DOPC, PC, PE and PS were from Sigma Aldrich and PtdIns(Ps) were from Echelon Biosciences. The surface was regenerated between runs by short pulses of 50 mM NaOH. The experiments were repeated using different preparations of liposomes and proteins.

### Dot Blot Overlay Assays

Dot blot overlay assays were performed according the protocol of the manufacturer (Echelon Biosciences), by incubating the fat-free milk blocked membranes with 10–50 µM 6xhis-tagged proteins. Bound proteins were detected using the primary anti 6His antibody (Roche), goat-anti mouse HRP conjugated secondary antibody and ECL detection.

### Spectrofluorimetric Measurements

Spectrofluorimetric measurements were performed at 25°C in 25 mM HEPES, 150 mM NaCl, pH 7.2 and 1 mM beta-mercaptoethanol in a Carry Eclipse Spectrofluorimeter (Varian). The peptides corresponded to the C-termini of syndecan-2 (TKEFYA) and usher (FTDTHL) and were of high purity (purity >95%, GeneCust), and the experiment were performed and analyzed as described previously [27]. Urea induced equilibrium unfolding experiments were performed using 1 µM of proteins (excitation 280 nm emission 320–380 nm) and the data fitted by a standard two-state model [60]. Unfolding reversibility was investigated by adding urea to solutions containing the native protein and by diluting the denaturant in solutions containing the denatured protein, which led to the same fraction of denatured and native protein, suggesting that the equilibrium unfolding was reversible.

The effects of the mutations on the stability were calculated by ΔΔ*G_D_*_−*N*_ = Δ*G_D_*_−*Nmut*_ − Δ*G_D_*_−*Nwt*_, where ΔΔG_D-N_ is the difference between the free energies of unfolding upon mutation.

**Structure based sequence alignments** made using ClustalX and Swiss pdb viewer.

## Supporting Information

Figure S1 **Related to** **Figure 1** **and** **2** **.** Control experiments showing that fluorescence intensities are comparable between constructs, fusions proteins are not cleaved or degraded, and showing the distribution of eYFP-PDZ constructs where S1PDZ1 enhancer is omitted.
(PDF)

Figure S2 **Related to** **Figure 2** **.** Confocal micrographs of eYFP-S1PDZZ-PDZX constructs in MCF-7, HEK293 and HeLa cell lines.
(PDF)

Figure S3 **Related to** **Figure 3** **.** Control microscopy experiments illustrating the effectiveness of lipid modifying treatments and showing additional examples of the effect or lack of effect of various treatments.
(PDF)

Figure S4 **Related to** **Figure 5** **.** Sequence alignment of PtdInsPs-interacting PDZ domains.
(PDF)

Table S1 **Related to** **Figure 4** **.** Oligomeric status of various recombinant PDZ domains used in this study.
(PDF)

Table S2 **Related to** **Figure 4** **.** Apparent K_D_ values for 19 recombinant PDZ domains for 5% PtdInsPs in DOPC liposomes.
(PDF)

Table S3 **Related to discussion.** Comparison of the localization of eYFP-S1PDZ1-PDZX domains and their host proteins based on Gene Ontology Annotations.
(PDF)

Table S4 **Related to Materials and Methods.** Amino-acid sequences of the PDZ domains used in this study.
(XLSX)
