# Supplementary material for: Prevalence, Specificity and Determinants of Lipid-Interacting PDZ Domains from an In-Cell Screen and In Vitro Binding Experiments
Source: PLoS One. 2013 Feb 4;8(2):e54581. doi: 10.1371/journal.pone.0054581 (PMC3563628; doi:10.1371/journal.pone.0054581)
Supplement: Figure S1 — Related to Figure 1 and 2 . Control experiments showing that fluorescence intensities are comparable between constructs, fusions proteins are not cleaved or degraded, and showing the distribution of eYFP-PDZ constructs where S1PDZ1 enhancer is omitted. (PDF) [file pone.0054581.s001.pdf]

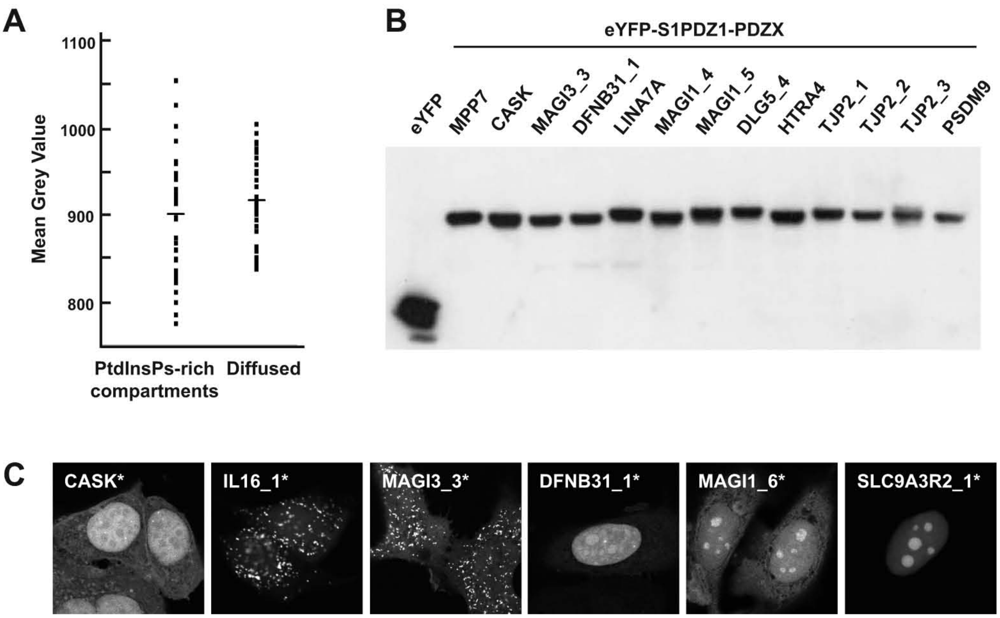

Supplementary figure 1.

Control experiments showing that eYFP-PDZ1S1 fusion constructs displayed comparable fluorescence intensities (A); were not degraded by proteolytic cleavage (B) and showing the distribution of various eYFP-PDZ constructs (C). **A.** Average fluorescence of MCF-7 cells overexpressing the eYFP-S1PDZ1-tagged PDZ domains. Each point represents one construct. Data for fusions concentrating in PtdInsPs-rich subcellular compartments (right) or with diffuse florescence (left) are compared. For each construct, three to five expressing cells were analyzed using wide-field fluorescence microscopy. Pictures were taken with exactly the same settings (source intensity, integration time, binning) and the average fluorescence of the delineated cells was quantified using the ImageJ software. The statistical significance was calculated using t-test comparing 35 constructs enriched in the PtdInsPs-rich subcellular compartments and 35 constructs localized diffusely. The average is indicated by the line and the p-value > 0.3 indicating no significant difference in the expression levels. **B.** Western blot showing the expression and integrity of various eYFP-S1PDZ1-PDZ fusions. Constructs with diffused localizations (LINA7A, MAGI1_4, MAGI1_5, DLG5_4, HTRA4, TJP2_1, TJP2_2, TJP2_3, PSDM9), and showing discrete plasma membrane (MPP7), strong plasma membrane (CASK), bright cytosolic spot (MAGI3_3), or subnuclear organelle (DFNB31_1) enrichment were selected at random, eYFP expressing cells were used as controls. To prepare total cell lysates, cell were washed twice with PBS and scraped on ice in lysis buffer (PBS supplemented with 1% NP40 and protease inhibitors). Next, samples were cleared by the centrifugation for 10 min, 16 000 g at 4 °C. For Western blot analysis, equivalent amount of the cell lysates were loaded on 4-12% gradient gels (Invitrogen) and the eYFP-S1PDZ1-PDZX constructs were detected using anti GFP antibody (1/1000) followed by Goat-anti-mouse-HRP antibody (1/10000). Signals were visualized by chemiluminescence detection reagent (Amersham Pharmacia Biotech). All constructs migrated at the expect molecular weight. **C.** Confocal micrographs of MCF- 7 cells transiently over-expressing eYFP-tagged PDZ domains when the PDZ1S1 enhancer is omitted (PDZ*).
