# Supplementary material for: Prevalence, Specificity and Determinants of Lipid-Interacting PDZ Domains from an In-Cell Screen and In Vitro Binding Experiments
Source: PLoS One. 2013 Feb 4;8(2):e54581. doi: 10.1371/journal.pone.0054581 (PMC3563628; doi:10.1371/journal.pone.0054581)
Supplement: Figure S2 — Related to Figure 2 . Confocal micrographs of eYFP-S1PDZZ-PDZX constructs in MCF-7, HEK293 and HeLa cell lines. (PDF) [file pone.0054581.s002.pdf]

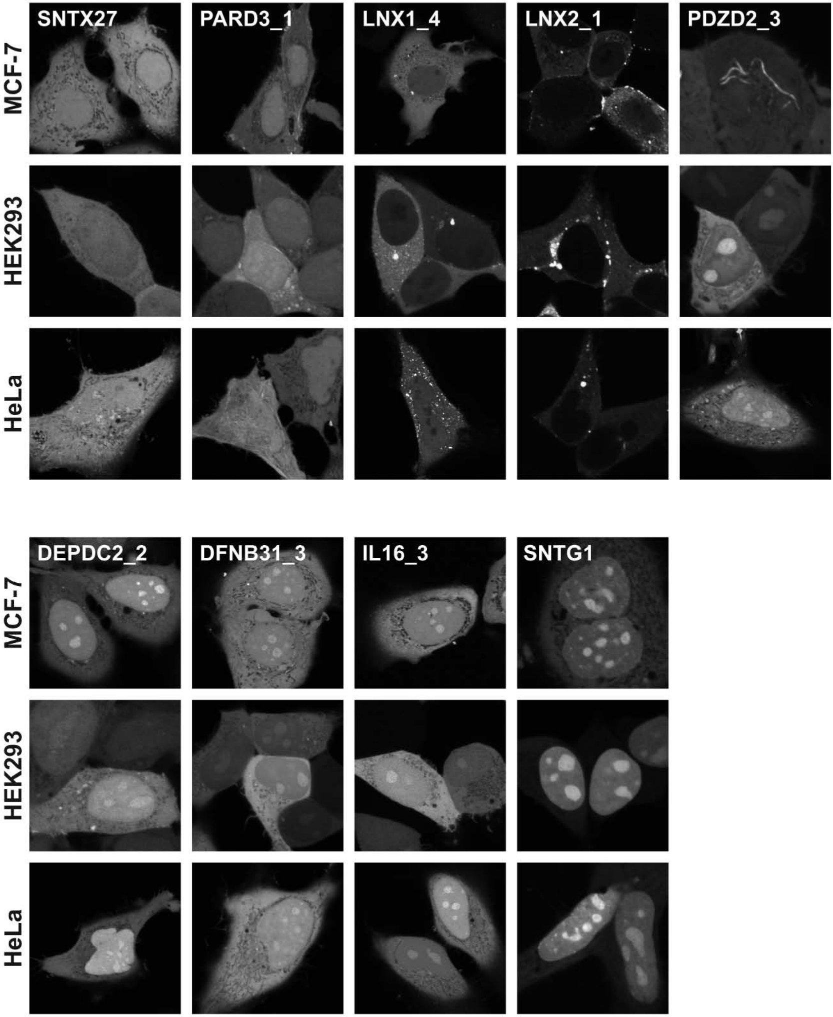

Supplementary figure 2.

Confocal micrographs of selected PDZ domains fused to eYFP-S1PDZ1 in MCF-7, HEK293 and HeLa cells as indicated.
