# Supplementary material for: Prevalence, Specificity and Determinants of Lipid-Interacting PDZ Domains from an In-Cell Screen and In Vitro Binding Experiments
Source: PLoS One. 2013 Feb 4;8(2):e54581. doi: 10.1371/journal.pone.0054581 (PMC3563628; doi:10.1371/journal.pone.0054581)
Supplement: Figure S3 — Related to Figure 3 . Control microscopy experiments illustrating the effectiveness of lipid modifying treatments and showing additional examples of the effect or lack of effect of various treatments. (PDF) [file pone.0054581.s003.pdf]

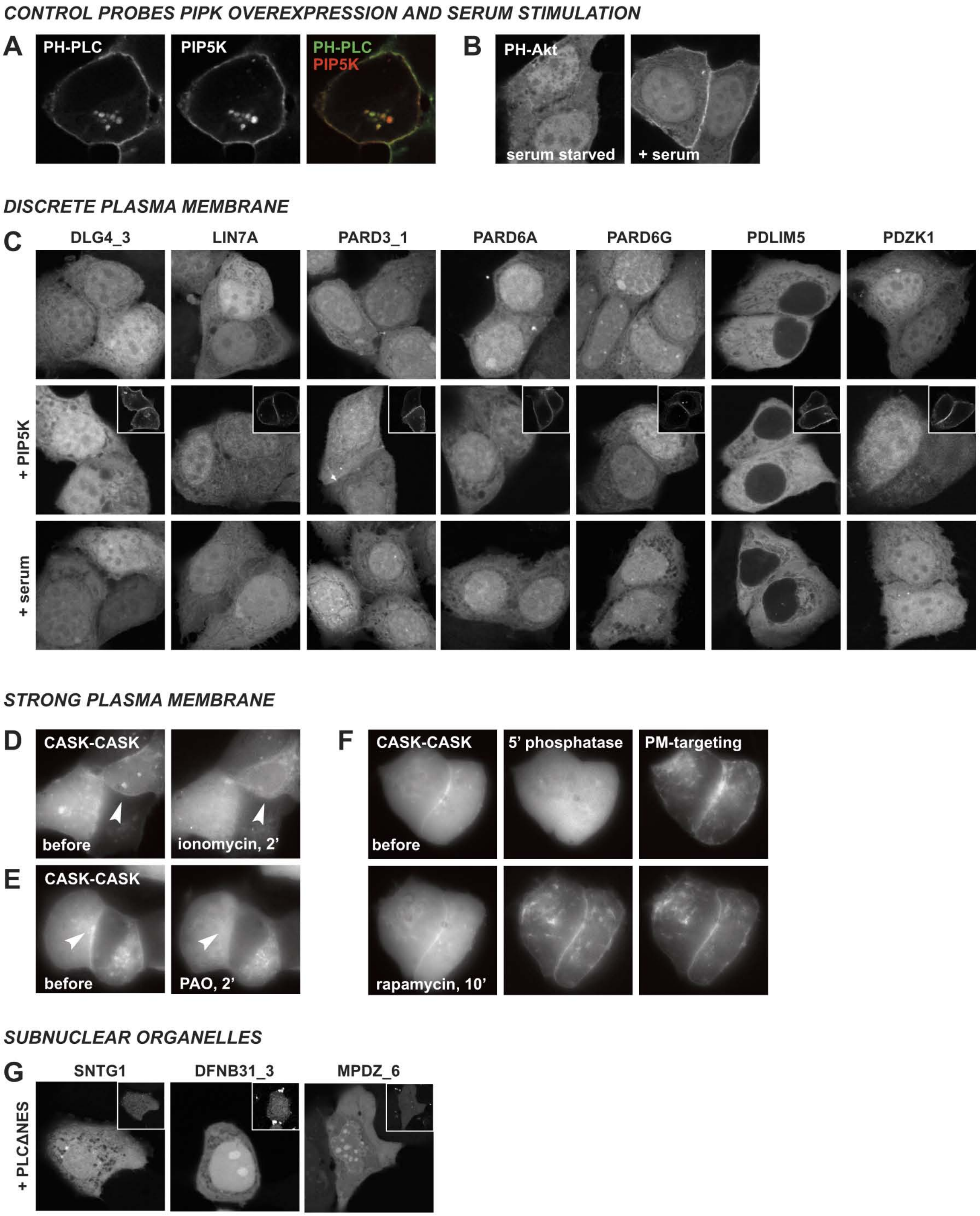

Supplementary figure 3.

Control fluorescence microscopy experiments in MCF-7 cells illustrating the effectiveness of the overexpression of PtdIns(4)P-5-kinase on the PtdIns(4,5)P2 probe, eYFP-PH-PLC (strong plasma membrane enrichment and colocalization) **(A)** and of serum stimulation on a PtdIns(3,4,5)P3 probe, eGFP-PH-Akt **(B)**. For quantitative data and further explanation see main text. **(C)** In contrast, these treatments have no effect on eYFP-PDZ1S1-PDZ fusion constructs showing discrete plasma membrane localization. PtdIns(4)P-5-kinase expression is shown in the insets. **(D-F)** Control experiments showing that the effect of various treatments affecting PtdIns(4,5)P2 levels have similar effect on eYFP-CASK-CASK than on eYFP-S1PDZ1-CASK (D,E,F to compare with E,F,G in main Figure 3). **(G)** Additional exemples illustrating the effect of mCherry-PLCΔNES (shown in the insets) on the subnuclear localization of eYFP-PDZ1-SNTG1(a relatively good *in vitro* PtdInsPs interactor) (for control image see Fig S1) and the lack of effect on the subnuclear localization of eYFP-PDZ1-DFNB31_3 and eYFP-PDZ1-MPDZ_6 (two poor PtdInPs interactors).
