# Supplementary material for: Prevalence, Specificity and Determinants of Lipid-Interacting PDZ Domains from an In-Cell Screen and In Vitro Binding Experiments
Source: PLoS One. 2013 Feb 4;8(2):e54581. doi: 10.1371/journal.pone.0054581 (PMC3563628; doi:10.1371/journal.pone.0054581)
Supplement: Figure S4 — Related to Figure 5 . Sequence alignment of PtdInsPs-interacting PDZ domains. (PDF) [file pone.0054581.s004.pdf]

Supplementary figure 4. Structure based sequence alignment of PtdInsPs interacting PDZ domains. Indicated in red are residues of the conserved positive charge cluster. Residues in blue indicate the alternative positive charge cluster of PICK1 (Pan et al., 2007). Basic residues in orange have been shown by mutagenic analysis to be of importance for SDCBPs PDZ-PtdInsPs interactions (Meerschaert et al., 2007; Mortier et al., 2005; Zimmermann et al., 2002). In magenta are residues defining a basic cluster in SLC9A3R2_1 potentially involved in PtdInsPs binding. Sequences of the predicted PtdInsPs binders APBA3-1, INADL_7, LIN7A and MPDZ_8 are included for comparison.

```
SLC9AR2_1       PRLCRLVRGEQ--GYGFHLHGEKGR------------R-GQFIRRVEP-GSPAEAAALRA
SNTX27          PRVVRIVKSES--GYGFNVRGQVSEGGQLRSINGELYAPLQHVSAVLPGGAADRAG-VRK
PDZD11          PRTITLKKPPGA-QLGFNIRGGKASQ------------LGIFISKVIPDSDAHRAG-LQE
DFNB31_1        VRLVSLRRAKAHEGLGFSIRGGSEHG------------VGIYVSLVEP-GSLAEKEGLRV
LIN7A           PRVVELPKTTD-EGLGFNVMGGKEQN------------SPIYISRIIPGGVAERHGGLKR
SNTG1           ERTVTIRRQTVG-GFGLSIKGGAEHN------------IPVVVSKISKEQRAELSGLLFI
SNTA1           RRRVTVRKADAG-GLGISIKGGRENK------------MPILISKIFKGLAADQTEALFV
LNX1_4          CKDIVLRRNTAG-SLGFCIVGGYEEYNG---------NKPFFIKSIVEGTPAYNDGRIRC
SDCBP2_2        QRTVTMHKDSMG-HVGFVIK--KG-----------------KIVSLVKGSSAARNG-LLT
SDCBP1_2        FETITMHKDSTG-HVGFIFK--NG-----------------KITSIVKDSSAARNG-LLT
IL16_1          ISNIVLMKGQAK-GLGFSIVGGKDSIYG---------PIGIYVKTIFAGGAAAADGRLQE
PDZD2_3         ISIIGLYKEKGK-GLGFSIAGGRDCIRG---------QMGIFVKTIFPNGSAAEDGRLKE
IL16_3          IHVTILHKEEGA-GLGFSLAGGADLEN-----------KVITVHRVFPNGLASQEGTIQK
MPDZ_8          LHMIELEKGHS--GLGLSLAGNKDRS-----------RMSVFIVGIDPNGAAGKDGRLQI
INADL_7         LHIIELEKDKN--GLGLSLAGNKDRS-----------RMSIFVVGINPEGPAAADGRMRI
SDCBP2_1        VREIHLCKDERG-KTGLRLRKVDQ---------------GLFVQLVQANTPASLVG-LRF
SDCBP1_1        IREVILCKDQDG-KIGLRLKSIDN---------------GIFVQLVQANSPASLVG-LRF
CASK            SRLVQFQKNTDE-PMGITLKMN---EL-----------NHCIVARIMHGGMIHRQGTLHV
MAGI1_6         FYTVELERGAK--GFGFSLRGGREYN------------MDLYVLRLAEDGPAERCGKMRI
PARD3_2         RLNIQLKKGTE--GLGFSITSRDVTIGG---------SAPIYVKNILPRGAAIQDGRLKA
PICK1           PGKVTLQKDAQN-LIGISIGGGAQYC------------PCLYIVQVFDNTPAALDGTVAA
DFNB31_3        STLVRVKKSAA--TLGIAIEGGANTR-----------QPLPRIVTIQRGGSAHNCGQLKV
MAGI3_3         LVTIPLIKGPK--GFGFAIADSP---------------TGQKVKMIL-D-S-QWCQGLQK
ZO1_2           PTKVTLVKSRKNEEYGLRLA------------------SHIFVKEISQDSLAARDGNIQE
APBA3_1         CREVHLEKRRG-EGLGVALVESGWGSLLP---------TAAVIANLLHGGPAERSGALSI
PARD3_1         VKLVEVPNDGG--PLGIHVVPFSARGGR---------TLGLLVKRLEKGGKAEHENLFRE

SLC9AR2_1       GDRLVEVNGVNVEGETHHQVVQRIKAV--EG---QTRLLVVD
SNTX27          GDRILEVNHVNVEGATHKQVVDLIRAG--EK---ELILTVLS
PDZD11          GDQVLAVNDVDFQDIEHSKAVEILKTA---R---EISMRVRF
DFNB31_1        GDQILRVNDKSLARVTHAEAVKALKGS---K---KLVLSVYS
LIN7A           GDQLLSVNGVSVEGEHHEKAVELLKAA---KD--SVKLVVRY
SNTG1           GDAILQINGINVRKCRHEEVVQVLRNA--GE---EVTLTVSF
SNTA1           GDAILSVNGEDLSSATHDEAVQVLKKT--GK---EVVLEVKY
LNX1_4          GDILLAVNGRSTSGMIHACLARLLKEL---KG--RITLTIVS
SDCBP2_2        NHYVCEVDGQNVIGLKDKKIMEILATA----GN-VVTLTIIP
SDCBP1_2        EHNICEINGQNVIGLKDSQIADILSTS----GT-VVTITIMP
IL16_1          GDEILELNGESMAGLTHQDALQKFKQA---KKG-LLTLTVRT
PDZD2_3         GDEILDVNGIPIKGLTFQEAIHTFKQI---RSG-LFVLTVRT
IL16_3          GNEVLSINGKSLKGTTHHDALAILRQAREPR---QAVIVTRK
MPDZ_8          ADELLEINGQILYGRSHQNASSIIKCA---PS--KVKIIFIR
INADL_7         GDELLEINNQILYGRSHQNASAIIKTA---PS--KVKLVFIR
SDCBP2_1        GDQLLQIDGRDCAGWSSHKAHQVVKKA---SGD-KIVVVVRD
SDCBP1_1        GDQVLQINGENCAGWSSDKAHKVLKQA---FGE-KITMTIRD
CASK            GDEIREINGISVANQTVEQLQKMLREEM--RG--SITFKIVP
MAGI1_6         GDEILEINGETTKNMKHSRAIELIKNG--GR---RVRLFLKR
PARD3_2         GDRLIEVNGVDLVGKSQEEVVSLLRST---KMEGTVSLLVFR
PICK1           GDEITGVNGRSIKGKTKVEVAKMIQEV---KG--EVTIHYNK
DFNB31_3        GHVILEVNGLTLRGKEHREAARIIAEAFKTKDRDYIDFLVTE
MAGI3_3         GDIIKEIYHQNVQNLTHLQVVEVLKQF--PVG-ADVPLLILR
ZO1_2           GDVVLKINGTVTENMSLTDAKTLIERS---KG--KLKMVVQR
APBA3_1         GDRLTAINGTSLVGLPLAACQAAVRETKS-QT--SVTLSIVH
PARD3_1         NDCIVRINDGDLRNRRFEQAQHMFRQAM--RTP-IIWFHVVP
```


Meerschaert, K., Bruyneel, E., De Wever, O., Vanloo, B., Boucherie, C., Bracke, M., Vandekerckhove, J., and Gettemans, J. (2007). The tandem PDZ domains of syntenin promote cell invasion. Exp Cell Res *313*, 1790-1804.

Mortier, E., Wuytens, G., Leenaerts, I., Hannes, F., Heung, M.Y., Degeest, G., David, G., and Zimmermann, P. (2005). Nuclear speckles and nucleoli targeting by PIP2-PDZ domain interactions. EMBO J *24*, 2556-2565.

Pan, L., Wu, H., Shen, C., Shi, Y., Jin, W., Xia, J., and Zhang, M. (2007). Clustering and synaptic targeting of PICK1 requires direct interaction between the PDZ domain and lipid membranes. EMBO J *26*, 4576-4587.
Zimmermann, P., Meerschaert, K., Reekmans, G., Leenaerts, I., Small, J.V., Vandekerckhove, J., David, G., and Gettemans, J. (2002). PIP(2)-PDZ domain binding controls the association of syntenin with the plasma membrane. Mol Cell *9*, 1215-1225.
