# Supplementary material for: Prevalence, Specificity and Determinants of Lipid-Interacting PDZ Domains from an In-Cell Screen and In Vitro Binding Experiments
Source: PLoS One. 2013 Feb 4;8(2):e54581. doi: 10.1371/journal.pone.0054581 (PMC3563628; doi:10.1371/journal.pone.0054581)
Supplement: Table S1 — Related to Figure 4 . Oligomeric status of various recombinant PDZ domains used in this study. (PDF) [file pone.0054581.s005.pdf]

Table S1. The state of purified recombinant His-tagged PDZ domains was assessed in different types of experiments until a conclusion was reached. SEC, size exclusion chromatography; CL, cross-linking; NG, Native Gel electrophoresis.

PDZ Domain	state	methods
DFNB31_1	Dimer with propensity to form oligomer.	SEC and CL
SCL9A3R2_1	Monomer in equilibrium with dimer/tetramer	SEC and NG
IL16_1	Dimer	SEC, NG and CL
PARD3_1	Monomer	SEC, NG and CL
MAGI1_6	Monomer	SEC
LNX1_4	Dimer with propensity to form oligomer.	CL
SNTG1	Monomer in equilibrium with dimer and oligomers	SEC and CL
CASK	Monomer	SEC, NG and CL
SNTX27	Monomer in equilibrium with dimer	SEC and CL
IL16_3	Monomer	SEC
MAGI3_3	Monomer	SEC
PDZD2_3	Monomer	SEC
PDZD11	Monomer	SEC
MPP7	Monomer	SEC
DFNB31_3	Propensity to form oligomer	CL
MPDZ_6	Monomer	SEC
MPDZ_7	Monomer	SEC
ERBB2IP	Monomer	SEC
SCRIB4	Monomer /Dimer + propensity to form oligomers	SEC and CL

SEC was directly performed on the purified proteins at 4°C on a Superdex 75 16/60 column (GE healthcare) in 25mM HEPES 150 mM NaCl pH 7.4 and 1mM β mercaptoethanol. Peak fractions were analyzed on 15% SDS PAGE gels. Native PAGE gels were 12%. For cross-linking experiments, 100 µg of protein in a total volume of 100 µl was treated with 5 µl of a 2.3 % freshly prepared solution of glutaraldehyde for 5 minutes at 37°C. The reaction was terminated by addition of 10 µl of 1 M Tris-HCl, pH 8.0. Cross-linked proteins were solubilized in loading buffer and separated by 12% SDS-polyacrylamide gel electrophoresis. State was determined after Western blotting with Ni-NTA HRP Conjugate antibody (Cat. no.34530 Qiagen) and ECL detection.
