# Supplementary material for: Prevalence, Specificity and Determinants of Lipid-Interacting PDZ Domains from an In-Cell Screen and In Vitro Binding Experiments
Source: PLoS One. 2013 Feb 4;8(2):e54581. doi: 10.1371/journal.pone.0054581 (PMC3563628; doi:10.1371/journal.pone.0054581)
Supplement: Table S2 — Related to Figure 4 . Apparent KD values for 19 recombinant PDZ domains for 5% PtdInsPs in DOPC liposomes. (PDF) [file pone.0054581.s006.pdf]

Table S2. Apparent K_D values (μM) for PtdInsPs-PDZ interactions as determined by SPR equilibrium analysis. Standard deviations are calculated from three independent experiments.

	PtdIns3P	PtdIns4P	PtdIns5P	PtdIns(3,4)P2	PtdIns(3,5)P2	PtdIns(4,5)P2	PtdIns(3,4,5)P2
**DFNB31_1**	16 ± 1	13 ± 5	14 ± 3	8.3 ± 2	5.8 ± 1	7.8 ± 2	3.9 ± 1
**SLC9A3R2_1**	>10	>10	>10	3.8 ± 0.9	5.3 ± 2	4.9 ± 1	4.8 ± 2
**IL16_1**	>10	>10	>10	5.9 ± 2	8.9 ± 2	4.3 ± 2	5.5 ± 2
**PARD3_1**	>20	>20	>20	6.3 ± 3	3.4 ± 1	8 ± 3	8.1 ± 1
**MAGI1_6**	>20	>20	>20	6.4 ± 2	8.5 ± 2	7.4 ±2	11 ± 4
**LNX1_4**	>20	>20	>20	13 ± 3	11 ± 5	14 ± 5	10 ± 5
**SNTG1**	> 100	46 ± 10	> 100	52 ±11	35 ± 11	22 ±3	12 ± 2
**CASK**	>100	>100	>100	34 ± 16	48 ± 15	25 ± 7	45 ± 20
**SNTX27**	65 ± 10	52 ± 15	60 ± 15	69 ± 10	50 ± 20	49 ± 20	34 ± 10
**IL16_3**	36 ± 11	32 ±8	23 ± 5	> 100	89 ± 40	76 ± 20	56 ±20
**MAGI3_3**	>70	> 70	> 70	28 ± 6	36 ± 15	47 ±14	32 ± 15
**PDZD2_3**	> 60	>60	>60	45 ± 13	29 ± 5	35 ± 8	39 ± 4
**PDZD11**	> 100	> 100	> 100	> 100	71 ± 25	56 ± 16	49 ± 10
**MPP7**	>40	>40	>40	>40	>40	>40	25 ± 8
**DFNB31_3**	>100	>100	>100	>100	86 ± 10	94 ± 13	67 ± 15
**MPDZ_6**	>100	>100	>100	>100	>100	>100	77 ± 30
**MPDZ_7**	>100	>100	>100	>100	>100	>100	80 ± 20
**ERBB2IP**	>100	>100	>100	>100	>100	>100	70 ± 20
**SCRIB_4**	> 100	> 100	> 100	> 100	> 100	> 100	> 100
