# Supplementary material for: Prevalence, Specificity and Determinants of Lipid-Interacting PDZ Domains from an In-Cell Screen and In Vitro Binding Experiments
Source: PLoS One. 2013 Feb 4;8(2):e54581. doi: 10.1371/journal.pone.0054581 (PMC3563628; doi:10.1371/journal.pone.0054581)
Supplement: Table S3 — Related to discussion. Comparison of the localization of eYFP-S1PDZ1-PDZX domains and their host proteins based on Gene Ontology Annotations. (PDF) [file pone.0054581.s007.pdf]

Table S3. Comparison between the cellular enrichments observed for the PDZ domains in the screening construct and the reported localizations of the host proteins based on Gene Ontology Annotations (GOA).

uniprot	Name	GOA	PDZ domain	Enrichment
Q02410	APBA1	synaptic vesicle	APBA1_1	Disrcete plasma membrane
			APBA1_2	Disrcete plasma membrane
O96018	APBA3	cytoplasm	APBA3_2	Disrcete plasma membrane
		membrane		
		perinuclear region of cytoplasm		
O14936	CASK	nucleus	CASK	Strong plasma membrane
		nuclear lamina		
		cytoplasm		
		plasma membrane		
		cell-cell junction		
		actin cytoskeleton		
		nuclear matrix		
		presynaptic membrane		
		cilium membrane		
		basement membrane		
Q70Z35	DEPDC2	intracellular	DEPDC2_2	Subnuclear organelles
Q8TB45	DEPDC6	intracellular	DEPDC6	Subnuclear organelles
				Bright cytosolic spots
Q9P202	DFNB31	photoreceptor inner segment	DFNB31_1	Subnuclear organelles
		stereocilia ankle link complex	DFNB31_3	Subnuclear organelles
		membrane fraction		
		soluble fraction		
		cytoplasm		
		actin filament		
		cilium		
		axon		
		dendrite		
		growth cone		
		photoreceptor connecting cilium		
		stereocilium		
		stereocilium bundle		
		cell projection		
		neuronal cell body		
		synapse		
Q12959	DLG1	basolateral plasma membrane	DLG1_2	Disrcete plasma membrane
		endoplasmic reticulum		
		nucleus		
		cytosol		

		immunological synapse<br>sarcolemma<br>tight junction<br>plasma membrane<br>postsynaptic density<br>cell junction<br>MPP7-DLG1-LIN7 complex		
Q15700	DLG2	plasma membrane<br>cytoplasm<br>postsynaptic membrane<br>cell junction<br>dendrite<br>neuronal cell body<br>synapse<br>juxtaparanode region of axon<br>postsynaptic density	DLG2_2	Disrcete plasma membrane
P78352	DLG4	cell junction<br>cortical cytoskeleton<br>dendrite cytoplasm<br>endocytic vesicle membrane<br>excitatory synapse<br>plasma membrane<br>postsynaptic membrane<br>synaptosome	DLG4_3	Disrcete plasma membrane
Q9C0E4	GRIP2	cytoplasm<br>plasma membrane	GRIP2_6	Subnuclear organelles
Q92743	HTRA1	extracellular region<br>cytoplasm	HTRA1	Disrcete plasma membrane
Q14005	IL16	extracellular region<br>intracellular<br>nucleus<br>cytoplasm<br>plasma membrane	IL16_1<br>IL16_3	Bright cytosolic spots<br>Subnuclear organelles
O14910	LIN7A	plasma membrane<br>tight junction<br>postsynaptic density<br>basolateral plasma membrane<br>synaptosome<br>cell junction<br>synapse<br>postsynaptic membrane	LIN7A	Disrcete plasma membrane
Q8TBB1	LNX1	cytoplasm	LNX1_4	Bright cytosolic spots

Q8N448	LNX2	NA	LNX2_1	Disrcete plasma membrane
				Bright cytosolic spots
			LNX2_4	Bright cytosolic spots
Q96QZ7	MAGI1	cytoplasm	MAGI1_6	Subnuclear organelles
		plasma membrane		
		cell-cell junction		
		tight junction		
		membrane		
		cell junction		
		cell projection		
Q86UL8	MAGI2	nucleus	MAGI2_4	Subnuclear organelles
		cytoplasm		
		plasma membrane		
		tight junction		
		postsynaptic density		
		cell junction		
		synaptosome		
		slit diaphragm		
		protein complex		
		synapse		
		perinuclear region of cytoplasm		
Q5TCQ9	MAGI3	intracellular	MAGI3_3	Bright cytosolic spots
		nucleus	MAGI3_6	Disrcete plasma membrane
		plasma membrane		
		tight junction		
P55196	MLLT4	nucleus	MLLT4	Disrcete plasma membrane
		cytosol		
		plasma membrane		
		cell-cell junction		
		adherens junction		
O75970	MPDZ	cytoplasm	MPDZ_6	Subnuclear organelles
		plasma membrane	MPDZ_7	Strong plasma membrane
		tight junction		
		postsynaptic density		
		apical plasma membrane		
		synaptosome		
		cell junction		
		dendrite		
		synapse		
		postsynaptic membrane		
Q00013	MPP1	membrane fraction	MPP1	Disrcete plasma membrane
		integral to plasma membrane		

		cortical cytoskeleton<br>stereocilium<br>cell projection		
Q9NZW5	MPP6	plasma membrane	MPP6	Disrcete plasma membrane
Q5T2T1	MPP7	adherens junction<br>tight junction<br>membrane<br>cell junction	MPP7	Disrcete plasma membrane
Q8TEW0	PARD3	cytosol<br>spindle<br>plasma membrane<br>cell-cell junction<br>adherens junction<br>tight junction<br>cell cortex<br>endomembrane system<br>cell junction<br>apical part of cell	PARD3_1	Disrcete plasma membrane<br>Subnuclear organelles
Q9NPB6	PARD6A	ruffle<br>nucleus<br>cytosol<br>plasma membrane<br>cell cortex<br>tight junction<br>cell junction<br>cell projection<br>apical part of cell	PARD6A	Disrcete plasma membrane
Q9BYG4	PARD6G	cytosol<br>plasma membrane<br>tight junction<br>cell junction	PARD6G	Disrcete plasma membrane<br>Subnuclear organelles
Q86UT5	PDZD3	cytosol<br>plasma membrane<br>brush border<br>subapical complex<br>apical part of cell	PDZD3_4	Disrcete plasma membrane
Q9H5P4	PDZD7	nucleus<br>cilium<br>cell projection	PDZD7_2	Bright cytosolic spots
Q5EBL8	PDZD11	extracellular region<br>cytosol<br>basolateral plasma membrane	PDZD11	Subnuclear organelles

Q5T2W1	PDZK1	cytoplasm<br>membrane<br>brush border membrane	PDZK1_1	Disrcete plasma membrane
Q9UPQ7	PDZRN3	neuromuscular junction	PDZRN3_1<br>PDZRN3_2	Disrcete plasma membrane<br>Disrcete plasma membrane
Q96SB3	PPP1R9B	protein phosphatase type 1 complex<br>nucleus<br>cytoplasm<br>cytoskeleton<br>plasma membrane<br>adherens junction<br>lamellipodium<br>cell junction<br>filopodium<br>ruffle membrane<br>cell projection<br>dendritic spine<br>synapse	PPP1R9B	Disrcete plasma membrane
Q8TEU7	RAPGEF6	intracellular<br>nucleus<br>cytoplasm<br>plasma membrane	RAPGEF6	Bright cytosolic spots
Q9H190	SCDBP2	intracellular<br>cytoplasm<br>plasma membrane	SCDBP2_1<br>SCDBP2_2	Subnuclear organelles<br>Subnuclear organelles
Q14160	SCRIB	cytoplasm<br>plasma membrane<br>cell-cell junction<br>adherens junction<br>membrane<br>Scrib-APC-beta-catenin complex<br>cell leading edge<br>postsynaptic membrane<br>presynaptic membrane	SCRIB_4	Subnuclear organelles
Q9Y566	SHANK1	membrane fraction<br>cytoplasm<br>plasma membrane<br>postsynaptic density<br>dendrite<br>dendritic spine<br>synapse<br>postsynaptic membrane	SHANK1	Disrcete plasma membrane<br>Subnuclear organelles

		excitatory synapse<br>ionotropic glutamate receptor complex		
O43166	SIPA1L1	intracellular<br>cytoplasm<br>cytoskeleton<br>plasma membrane<br>postsynaptic density<br>synaptosome<br>cell junction<br>dendritic spine<br>synapse<br>postsynaptic membrane	SIPA1L1	Subnuclear organelles
O14745	SLC9A3R1	ruffle<br>cytoplasm<br>centrosome<br>plasma membrane<br>microvillus<br>endomembrane system<br>actin cytoskeleton<br>apical plasma membrane<br>filopodium<br>brush border membrane<br>microvillus membrane<br>cell projection<br>intracellular membrane-bounded organelle<br>nucleus<br>cell periphery	SLC9A3R1_1	Subnuclear organelles
Q15599	SLC9A3R2	nucleus<br>cytoplasm<br>endomembrane system<br>plasma membrane<br>apical plasma membrane	SLC9A3R2_1<br>SLC9A3R2_2	Subnuclear organelles<br>Subnuclear organelles
Q9NSN8	SNTG1	nucleus<br>cytoplasm<br>cytoskeleton<br>syntrophin complex<br>ruffle membrane	SNTG1	Subnuclear organelles
Q9UMS6	SYNPO2	nucleus<br>cytoplasm<br>actin cytoskeleton<br>Z disc	SYNPO2	Disrcete plasma membrane
O95049	TJP3	plasma membrane<br>tight junction	TJP3_1	Subnuclear organelles

	apical plasma membrane
	cell junction
